# Comprehensive promoter level expression quantitative trait loci analysis of the human frontal lobe

**DOI:** 10.1186/s13073-016-0320-1

**Published:** 2016-06-10

**Authors:** Cornelis Blauwendraat, Margherita Francescatto, J. Raphael Gibbs, Iris E. Jansen, Javier Simón-Sánchez, Dena G. Hernandez, Allissa A. Dillman, Andrew B. Singleton, Mark R. Cookson, Patrizia Rizzu, Peter Heutink

**Affiliations:** Applied Genomics for Neurodegenerative Diseases, German Center for Neurodegenerative Diseases (DZNE), Tübingen, Germany; Genome Biology of Neurodegenerative Diseases, German Center for Neurodegenerative Diseases (DZNE), Tübingen, Germany; Laboratory of Neurogenetics, National Institute on Aging (NIA), Bethesda, Maryland USA; Department of Molecular Neuroscience, Institute of Neurology, University College London, London, UK; Department of Clinical Genetics, VU University Medical Center (VUmc), Amsterdam, The Netherlands; Department of Neurodegenerative Diseases, Hertie Institute for Clinical Brain Research, University of Tübingen, Tübingen, Germany

**Keywords:** Expression quantitative trait loci (eQTL), frontal lobe cortex, NRGN, Cap analysis gene expression sequencing (CAGEseq), PARK16

## Abstract

**Background:**

Expression quantitative trait loci (eQTL) analysis is a powerful method to detect correlations between gene expression and genomic variants and is widely used to interpret the biological mechanism underlying identified genome wide association studies (GWAS) risk loci. Numerous eQTL studies have been performed on different cell types and tissues of which the majority has been based on microarray technology.

**Methods:**

We present here an eQTL analysis based on cap analysis gene expression sequencing (CAGEseq) data created from human postmortem frontal lobe tissue combined with genotypes obtained through genotyping arrays, exome sequencing, and CAGEseq. Using CAGEseq as an expression profiling technique combined with these different genotyping techniques allows measurement of the molecular effect of variants on individual transcription start sites and increases the resolution of eQTL analysis by also including the non-annotated parts of the genome.

**Results:**

We identified 2410 eQTLs and show that non-coding transcripts are more likely to contain an eQTL than coding transcripts, in particular antisense transcripts. We provide evidence for how previously identified GWAS loci for schizophrenia (*NRGN*), Parkinson’s disease, and Alzheimer’s disease (PARK16 and *MAPT* loci) could increase the risk for disease at a molecular level. Furthermore, we demonstrate that CAGEseq improves eQTL analysis because variants obtained from CAGEseq are highly enriched for having a functional effect and thus are an efficient method towards the identification of causal variants.

**Conclusion:**

Our data contain both coding and non-coding transcripts and has the added value that we have identified eQTLs for variants directly adjacent to TSS. Future eQTL studies would benefit from combining CAGEseq with RNA sequencing for a more complete interpretation of the transcriptome and increased understanding of eQTL signals.

**Electronic supplementary material:**

The online version of this article (doi:10.1186/s13073-016-0320-1) contains supplementary material, which is available to authorized users.

## Background

Genome wide association studies (GWAS) for neurological and neuropsychiatric conditions have successfully identified DNA loci associated with the risk of developing disease [1]. These loci provide attractive starting points to improve our understanding of the molecular processes underlying disease, in particular given that Mendelian and sporadic forms often share common risk factors. In Parkinson’s disease (PD), for example, rare mutations in the genes *SNCA* and *LRRK2* cause familial PD [2, 3], while common genetic variation in or near these genes is associated with a risk for the common non-familial forms of the disease [4]. While mutations identified for Mendelian disorders generally occur in protein-coding genes, the large majority of GWAS risk loci are located in non-coding or poorly annotated regions, making the interpretation of their role in disease etiology challenging [5]. In order to identify the causal variant(s) underlying GWAS peaks, it is therefore essential to generate more targeted data to identify the biological consequences of genomic variants.

Recently, collaborative efforts such as ENCODE and FANTOM5 have provided evidence that a large proportion of the non-coding genome is transcribed, harbors elements that regulate gene expression, and has a biological function [6, 7]. It is thus plausible that a large proportion of the GWAS loci detect the effect of risk variants within non-coding regulatory DNA elements, which can be located at a considerable distance from protein-coding and non-coding genes. The identification of these regulatory variants and their associated genes and transcripts may be helpful in understanding GWAS findings and to establish the exact relationships between variation, genes, and disease. An additional difficulty in the interpretation of GWAS results is that associated risk loci often span a considerable genomic region, containing a large number of variants distributed over multiple genes, transcripts, and regulatory elements. Correlation of transcript expression levels with genomic variants or quantitative trait loci (eQTL) analysis is a powerful tool to explore the possible biological consequences of candidate GWAS variants in the associated region and it can help to limit the number of variants to be considered as possibly causal.

Most eQTL studies have focused on easily obtainable cell types, such as lymphoblast cell lines or fibroblasts, but it has been shown that a considerable proportion of eQTLs are cell-specific, tissue-specific, and even brain region-specific [8–10]. This is supported by data generated from the FANTOM5 project, showing that the transcriptome of the human brain is complex and that many transcripts that are unique to the brain exist [6]. In addition, the FANTOM5 project provides clear evidence that enhancer elements often function in a cell-specific or tissue type-specific manner [11]. It is therefore essential to generate eQTL data on the appropriate tissue and cell types for neurological and neuropsychiatric disease [8, 10]. Laser capture microscopy to isolate pure populations of a single cell type from human brain postmortem tissues is currently not feasible as it is not possible to isolate complete neurons with their complex structure of axons and dendrites. Isolating postmortem brain tissue samples cannot reliably determine the expression patterns of the cell types present in the sample, but it has the advantage of capturing the complex interplay of expression patterns between the different cell types present in different brain regions.

Although several eQTL studies on human postmortem brain tissues have been performed, the majority of them are based on microarrays, which are often limited to existing gene annotations of protein-coding transcripts and a small number of non-coding genes [9, 12–14]. These arrays do not capture the very large diversity of RNAs that are known to be present in the human brain and in large part will not capture diverse transcription start sites at a single gene. These include messenger RNAs (mRNAs) emerging from brain-specific alternative promoters and brain-specific non-coding RNAs [6, 7, 15]. Thus, it is necessary to use complementary experimental methods that are not biased by existing annotation and that can probe for these features.

The frontal lobe performs a multitude of functions related to planning behavioral responses to external and internal stimuli and is involved in speech, emotions, and problem-solving (see ref [16] for an overview). In addition, the frontal cortex has been associated with important neurological and neuropsychiatric diseases, such as frontotemporal dementia, autism, and schizophrenia. To create a detailed characterization of the frontal cortex transcriptome, we generated gene expression data using cap analysis gene expression (CAGE) combined with next generation sequencing (CAGEseq) on RNA isolated from postmortem samples of the frontal cortex of 128 individuals with no clinical signs of neurological disorders. CAGE was first introduced in 2003 [17], later improved for identification of non-polyadenylated RNAs [18] and a protocol for next generation sequencing became available in 2012 [19]. CAGE captures all 5′ ends of capped RNA transcripts and subsequent sequencing results in short (usually 20 or 27 bp) reads representing mainly transcription start sites (TSS).

Matching genotype data were generated using a combination of whole-genome genotyping arrays, exome sequencing, and variant calling from our CAGEseq data. CAGEseq provides high resolution strand-specific profiling of TSS in a quantitative and annotation independent manner and allows for the identification of coding, non-coding, and novel transcript, as well as overlapping genes transcribed from opposite strands. In addition, genomic variants identified with CAGEseq are located in the immediate vicinity of TSSs and sequences of core promoters and are therefore likely to have a functional effect.

Our data allowed us to build a detailed transcriptional map of the frontal lobe and to identify a multitude of new transcripts and TSSs. We subsequently used these data to perform eQTL analysis and identified 168,588 CAGE-cluster–genotype pairs altering the transcription of 2410 unique CAGE-clusters. In those TSSs influenced by a *cis*-eQTL we show enrichment in non-coding RNAs compared to protein-coding genes. By cross-referring published GWAS loci for neurological and neuropsychiatric diseases, we identified several GWAS loci that are associated with altered expression of genes. Our findings may help to elucidate some of the molecular mechanisms underlying the associated risk factors for these disorders.

## Methods

### Collection and RNA isolation of postmortem brain tissue

Frozen human frontal lobe material was collected from 128 neurologically normal individuals. Sample collection consisted of 90 males and 39 females with a mean age of death of 51 years (range, 2–95 years) and a mean postmortem interval (PMI) of 11 h (range, 1–28 h). Total RNA was extracted from the frontal lobe of each individual using Life Technologies TRIzol. RNA quality was measured using the Agilent 2100 Bioanalyzer and 2200 TapeStation. On average samples had an RNA integrity number (RIN) of 7.7 (range, 5.4–9.1). The use of human brain samples was approved by the NIH Office for Human Subjects Research. A complete list of the included samples is available in Additional file 1: Table S1.

### Genotype data

Single nucleotide polymorphism (SNPs) and indels, collectively described as variants, were generated using three different platforms.

#### SNP arrays

Genome-wide tagging SNPs were genotyped using Illumina Infinium HumanHapmap550 or Human610 BeadChip for all individuals. Genotypes were filtered using PLINK (version 1.07) [20] with the following quality control cut off values: individuals were excluded when the call rate was lower than 95 % and SNPs with a minor allele frequency (MAF) below 5 %; a missing rate per SNP above 5 %; or with a Hardy-Weinberg equilibrium (HWE) test *p* value of < 1e-6 were removed. Genotype data were used to estimate cryptic relatedness between individuals. No individuals were found to be closely related at a pihat threshold of 0.05. Multidimensional scaling (MDS) plot including HapMap (Phase 2 release 23) [21] populations revealed one individual with African ancestry, which was removed for subsequent analysis. See Additional file 2: Figure S1 for the MDS plot including HapMap populations.

Imputation was performed with MaCH [22] and MiniMac [23] based on the European reference haplotype from the 1000 Genomes Phase1 v2.20101123 data [24]. Prior to imputation, genotyped SNPs were filtered to remove variants where the call rate was less than 95 %, the minor allele frequency was less than 1 %, and the HWE *p* value was of <1e-6. Post imputation, any variant where the imputation quality score was less than 0.3 (r^2^ from MiniMac) was excluded from analysis.

#### Exome-sequencing variants

Illumina Truseq version 2 exome-sequencing of 102 individuals was already performed for another study in parallel by the North American Brain Expression Consortium (NABEC). Exome enrichment libraries were prepared according to the standard Illumina exome capture protocol. Paired-end sequence reads were aligned using Burrows-Wheeler Aligner (BWA) [25] against the human reference genome (hg19). The Picard toolset was used for format conversion, sorting, indexing, and duplicate read identification of the aligned reads. The Genome Analysis Toolkit (GATK) [26, 27] was used to recalibrate base scores, perform local re-alignments around indels, and to call and filter variants based on the GATK version 3 best practices. Variants with a missing rate above 5 %, a MAF below 5 %, or with a HWE test *p* value <1e-6 were removed.

#### CAGEseq variants

In order to obtain variation information in the vicinity of TSSs, variants were called from our CAGEseq data using SAMtools (version 0.1.18) [28] and VARSCAN (version 2.3.6) [29]. Only variants with a minimal coverage of 20×, a minimal average quality of 20, a minimum variant allele CAGEseq read frequency of 0.25, and a minimum frequency to call homozygote of 0.73 were selected. Variants were annotated and filtered based on the presence in dbSNP138 database using ANNOVAR [30] and filtered in PLINK using a missing rate above 5 %, a MAF below 5 %, or with a HWE test *p* value <1e-6 were removed. When CAGEseq variants were identified as eQTL, expression of both alleles was confirmed by visual inspection to prevent allele specific expression.

#### Merged dataset

Variants from the three aforementioned datasets were merged. For those variants present in more than one dataset, genotypes were assigned based on the following ranking: BeadChip SNPs > exome sequencing variants > CAGEseq-derived variants > imputed variants. Concordance between variants called in different platforms was generally high (>90 % see Additional file 2: Table S2). In total 5,729,884 variants were left for analysis. Exome-sequencing, BeadChip, and CAGEseq variants datasets were confirmed to represent the same individual based on overlapping variants. All variants were annotated using ANNOVAR [30] to identify genetic characteristics.

### Gene expression data analysis

#### Library preparation

Libraries were constructed using a published CAGEseq protocol adapted for next generation sequencing [19]. Briefly, complementary DNA (cDNA) was synthesized from total RNA using 15 nucleotides long random primers. This process was carried out in the presence of trehalose and sorbitol to extend cDNA synthesis through GC-rich regions in 5′ untranslated regions (UTR). The 5′ ends of messenger RNA within RNA-DNA hybrids were selected by the cap-trapper method [18] and ligated to a linker so that an EcoP15I recognition site was placed adjacent to the start of the cDNA, corresponding to the 5′ end of the original messenger RNA. This linker was used to prime second-strand cDNA synthesis. Subsequent EcoP15I digestion released the 27-base pairs (bp) CAGEseq tags. After ligation of a second linker, CAGEseq tags were PCR-amplified, purified, and strand-specific sequenced on the Illumina HiSeq 2000 for 50 bp single end reads.

#### Sequencing data preprocessing and quality control

CAGEseq data were processed using a previously described analysis pipeline [15]. Briefly, Illumina reads were demultiplexed and trimmed using FASTX toolkit (hannonlab.cshl.edu/fastx_toolkit/). Then CAGEseq reads were filtered for artifacts using TagDust (version 1.12) [31] and mapped to the human genome (hg19) using BWA (version 0.5.9) for short reads (aln and samse commands) [25]. Gender was confirmed based on X-inactive specific transcript (*XIST*) expression and thereafter reads mapping to chromosomes X, Y, and M were removed to minimize gender and normalization biases.

Mapped CAGEseq reads were grouped into CAGE-clusters using a series of Python scripts designed at the RIKEN Omics Science Center [32]. In brief, single base pair promoters were produced by determining all positions in the genome to which the 5' end of at least one CAGEseq read was mapped, excluding reads with a mapping quality lower than 20, which results in the exclusion of multimapping reads. Single base pair promoters within 20 bp of each other were merged into one CAGE-cluster and raw counts were normalized dividing the number of CAGEseq reads observed at a given CAGE-cluster by the total number of mapped tags in the library and multiplied by 1 million (tags per million, tpm).

MDS plot of the logged normalized expression values at these clusters was inspected for the identification of outliers. This analysis led us to the removal of eight samples, of which six had a low amount of CAGEseq sequence reads (<1 million) and two had a low 5′UTR mapping rate (<25 %) indicating low quality libraries (see Additional file 2: Figure S2 and S3 for the MDS plots before and after removal, respectively). After removing these sample outliers, only CAGE-clusters with expression of at least 5 tpm in at least one sample and a signal detectable in at least 10 % of the samples were included in subsequent analyses. The final dataset contained a total of 27,476 CAGE-clusters.

#### Annotation and visualization

Annotation of the identified CAGE-clusters was performed using GENCODE version 17 using the following mapping categories: TSS, exon, intron, pseudogene (all sense or antisense), and intergenic [33]. In addition, we classified the CAGE-clusters in terms of GENCODE transcript classes (coding, non-coding, retained intron, intergenic) and biotypes (protein-coding, processed transcript, intergenic, nonsense mediated decay, retained intron, lincRNA, antisense). CAGE-clusters were named using the gene symbols they mapped to and a number indicating their rank based on expression level in the corresponding gene. For visualization of the CAGEseq data, we used the ZENBU omics data integration and interactive visualization system [34].

### Expression quantitative trait loci analysis

eQTL analysis was performed using Matrix eQTL (version 2.1.0) [35]. Standard linear regression was performed for each variant against every identified CAGE-cluster using log-transformed CAGE-cluster expression values assuming an additive affect.

To prevent confounding effects, we included in the model four known covariates (age, gender, RIN value, and PMI) and additionally the first six principal components resulting from principal component analysis (PCA) performed on the expression data with the four known covariates regressed out (see Additional file 2: Figure S4). Significance threshold was set as false discovery rate (FDR) <1 % (results are reported in Additional file 2: Tables S3 and S6), calculated using Benjamini–Hochberg procedure implemented in Matrix eQTL (assumes all SNP-gene pairs tested are independent). Each CAGE-cluster was tested for association with every variant *in cis* – defined as a range of 1 Mb upstream or downstream of the identified CAGE-cluster—or *in trans* effect—defined as more than 1 Mb upstream or downstream of the identified CAGE-cluster or on other chromosomes. Results were separated into three lists: one containing all *cis* variants; a *cis* eQTL sentinel list containing the highest associated variant per CAGE-cluster (in case of multiple variants with the same *p* value, the closest to the CAGE-cluster was chosen); and one with all *trans* variants. For all sentinel *cis* eQTL variants, RegulomeDB scores were obtained to assess whether variants might affect transcription factors binding [36].

### External datasets

#### Annotation of intergenic CAGE-clusters

Validation of intergenic CAGE-clusters was performed by intersection with other public datasets. The following datasets were used: (1) RefSeq genes (downloaded from UCSC genome browser, last updated 2014-08-19), to identify genes absent from GENCODE v17; (2) FANTOM5 phase I permissive TSSs, to verify whether the intergenic peaks were consistent between CAGEseq experiments (downloaded from ZENBU); (3) Repetitive Elements (downloaded from UCSC genome browser, updated 2009-04-24), since expression can arise from repeats [37]; (4) a recently published CAGEseq expression derived enhancer dataset [11]; and (5) frontal cortex H3K4me3 ChIP-Seq data [38]. ChIP-Seq peak calling was performed on each sample using MACS (version 1.4.2) with parameters –bw = 230 and -tsize 36 and using the input controls available in the original data [39]. A pool of 41,091 ChIP-Seq peaks was created considering all ChIP-Seq peaks called in at least one sample and merging adjacent peaks.

#### Replication of eQTL variants and GWAS catalog intersection

Replication was sought between our identified eQTL variants and previously published eQTL studies in brain and other tissues (listed in Additional file 2: Table S3). For this, we determined the overlap between our list of eQTL variants and published ones and evaluated for the RNA sequencing (RNA-seq) eQTL data whether the overlapping ones influenced the expression of the same gene/transcript. Additionally, we overlaid our results with the GWAS catalog, containing genomic locations associated with disease from 1927 studies and containing over 14,000 variants (retrieved August 2014) [1]. To assess enrichments in eQTL variants genomic locations, we used as genome average the set of all included variants. We found that the local linkage disequilibrium structures were highly similar between the set of all included variants and sentinel variants (Additional file 2: Figure S5). In the MAF distribution, there appears to be some differences between the set of all included variants and sentinel variants (Additional file 2: Figure S6). More precise enrichment estimates could be obtained matching on this property in the null distribution.

#### Functional elements enrichment in expression quantitative trait loci variants

To test whether the identified eQTL variants were located in genomic regions with regulatory function, we intersected eQTL variant locations with H3K27ac (histone modification often found near active regulatory elements) and DNase Hypersensitivity Sites (DHS, mapping to open accessible chromatin) data. Two frontal lobe H3K27ac ChIP-seq libraries, each representing uniquely mapped reads after duplicate removal, were downloaded from the Roadmap Epigenomics Project genome browser (retrieved 20/05/2014). Peak calling was performed for each library independently using MACS [39] with default parameters.

Two DHS datasets produced in the context of the ENCODE project were downloaded from UCSC genome browser. A first dataset, that we named DHS-general, represents aggregated data from 125 cell lines (downloaded from UCSC genome browser; retrieved 02/07/2014). The second, which we named DHS-brain, is limited to frontal cerebrum and frontal cortex samples (downloaded from UCSC genome browser, retrieved 14/04/2014). The set of DHS peaks used for the intersection represents the union of the two datasets (151,372 peaks).

#### MiTranscriptome database

Supportive evidence for gene model structures was sought using a public RNA-seq database recently created named MiTranscriptome (http://www.mitranscriptome.org) [40]. In ZENBU, a browser track of the MiTranscriptome assembly was present, based on the bed file available at http://www.mitranscriptome.org. At regions of interest (e.g. intergenic CAGE-clusters) this track was used to identify the potential transcript structure and the DNA sequence of these transcripts was used for PCR primer design.

### Experimental expression quantitative trait loci variant validation

To validate the intragenic NRGN_TSS4 cluster and therefore corroborate the identified eQTL, we isolated total RNA from frontal cortex from six additional donors. Samples were selected from our internal CAGEseq expression data based on their NRGN_TSS4 expression: three samples (indicated as donors 1, 3, and 4 in Additional file 2: Table S4) showed expression for NRGN_tss4 and three samples (donors 2, 5, and 6) showed no expression. Primers were designed directly in the NRGN_tss4 region and in the exon 2 of *NRGN* (Additional file 2: Table S5). Total RNA primed with oligoDT and random hexamers was used for cDNA synthesis with Life Technologies Superscript III according to the manufacturer protocol. PCRs reactions with the cDNA as template were performed to validate the NRGN_TSS4 and the structure of the neurogranin (*NGRN*) transcript initiating at NRGN_TSS4. Amplified bands were Sanger sequenced using Life Technologies BigDye terminators chemistry v3.

To verify the structure of the potential new antisense transcript at the PARK16 locus, PCR reactions were performed on cDNA and the amplified band was sequenced as described above. Primers were designed directly in the identified eQTL CAGE-cluster region and in the nearest exon predicted by the MiTranscriptome database. Primer sequences and PCR conditions for both *NRGN* and PARK16 are provided in Additional file 2: Table S5. Genotyping of rs35306015 on DNA from donors 1 to 6 and rs320881 of three additional samples already used in the FANTOM5 brain CAGEseq libraries was performed by PCR amplification and subsequent Sanger sequencing as described above (see Additional file 2: Table S6 for primers sequences).

### Intersections and plots

Intersections between variants and supporting datasets were performed using BEDtools suite (version v2.17.0) [41]. Intersections with DHS data were performed using windowBed and 500 bp added upstream and downstream (-w 500); intersections with H3K27ac with 50 bp pairs added upstream and downstream (-w 50), chosen consistently with FANTOM5 [6]. The *NRGN* Spearman correlation plot was generated based on expression values of all *NRGN* locus CAGE-clusters using R. One sample (UMARY-1027) was excluded from correlation plot due to low *NRGN* expression. All plots and statistical calculations were performed using R (https://www.r-project.org/).

## Results and discussion

### Frontal lobe transcriptome

**Fig. 1:**
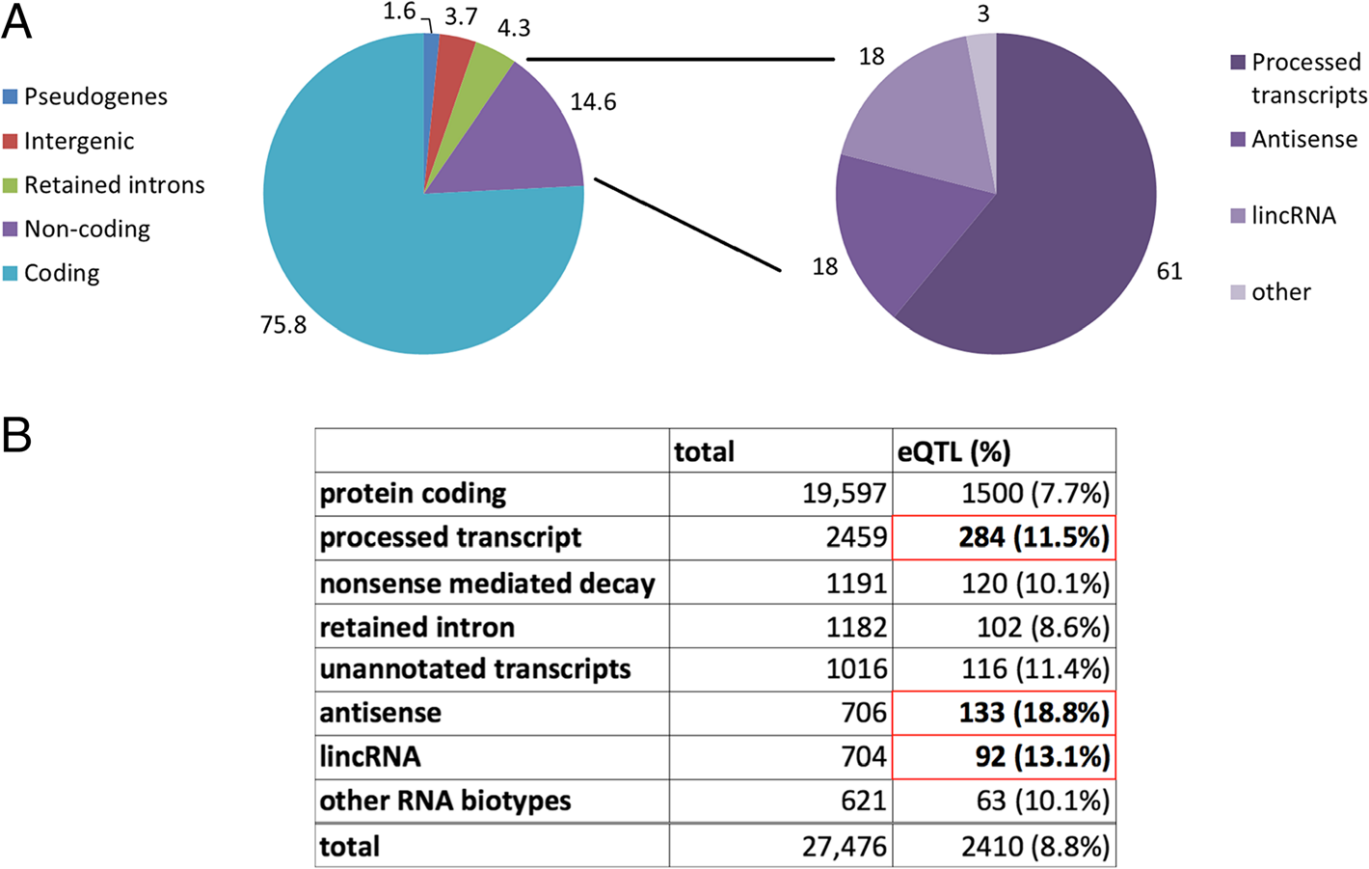
Frontal lobe transcriptome overview with expression features and eQTL percentages. **a** *Pie chart* showing the relative contributions (in %) of the different GENCODE version 17 transcript classes and biotypes to the overall expressed CAGE-clusters (*left*) and a breakdown of the non-coding transcripts in subtypes (*right*). **b** Percentage of CAGE-clusters per biotype class identified as eQTL: 7.7 % of the total identified protein-coding transcripts were identified as eQTL, while the percentage of non-coding transcripts eQTLs (within the *red boxes*) is significantly higher especially for antisense transcripts

We generated more than 1.5 billion CAGEseq reads (average 12.5 million per sample) that mapped to the human reference genome from 119 frontal lobe samples of neurologically normal individuals. After preprocessing and normalization, CAGEseq reads were grouped into 27,476 CAGE-clusters representing putative TSSs and mapping to 15,324 distinct genes. Of the 27,476 CAGE-clusters, the majority mapped into known TSSs regions (>71 %) and overlapped with a FANTOM5 identified TSSs (>74 %). Protein-coding transcripts accounted for the majority (>71 %) of CAGE-clusters. The remaining clusters represent non-coding transcripts of different classes including 1016 non-annotated intergenic CAGE-clusters. A detailed feature annotation is presented in Fig. 1a and 1b.

To corroborate that the 1016 non-annotated intergenic CAGE-clusters are genuine, we used five publically available datasets. We found supportive evidence for 52 % of the intergenic CAGE-clusters of being associated with H3K4me3 marks, 51 % with known repeat elements, 20 % overlapped with a TSS of the FANTOM5 permissive set, 10 % with CAGE-defined enhancers, and 7 % with a RefSeq gene (note that each CAGE-cluster can overlap any of the datasets and therefore percentages are not expected to add to 100 %). Overall, 89 % of the intergenic CAGE-clusters are likely to be genuinely transcribed. (See Additional file 3: Table S12 for a full list of all identified TSS and annotation and Additional file 4: Table S13 for re-annotation of the intergenic peaks). For the remaining 11 % (182 CAGE-clusters, 0.7 % of total identified CAGE-clusters), no functional domain could be identified, indicating they are new transcripts, unknown functional elements, or technical artifacts.

### eQTL discovery and replication

After combining 477,872 Illumina BeadChip SNPs, 81,397 exome derived common variants (including 5037 indels), 930 CAGEseq SNPs, and 5,240,393 imputed variants, removing duplicates and filtering all genotyping datasets, a total of 5,729,884 common variants remained for eQTL analysis. We then searched for eQTL associations using an additive linear model considering a region of 1 Mb upstream and downstream of the identified CAGE-cluster for *cis* effects and distances greater than 1 Mb for *trans* eQTLs.

#### Cis eQTL discovery

By using a FDR of 1 %, we identified 141,468 unique variants influencing 2410 unique CAGE-clusters *in cis* (8.8 % of total CAGE-clusters expressed in our dataset) representing 2113 distinct genes. On average, 1.19 CAGE-clusters (range, 1–5) were influenced by a single variant, while we identified on average 69.9 variants (range, 1–1137) per CAGE-cluster. Of the *cis* influenced transcripts 62.2 % (n = 1500) were coding, 21.2 % (n = 509) were non-coding, 4.8 % (n = 116) were intergenic, and 11.8 % (n = 285) mapped to retained introns, nonsense mediated decay, or other RNA biotypes. The 509 non-coding transcripts consisted of 284 processed transcripts (55.8 %), 133 antisense transcripts (26.1 %), and 92 lincRNAs (18.1 %) (Fig. 1b and Additional file 3: Table S12). Among the 116 intergenic CAGE-clusters that were influenced by an eQTL, 84 % were supported by other datasets (ChIP-seq peaks (65 %), repeat element (43 %), FANTOM5 identified TSS (25 %), transcript in the RefSeq database (5 %), and CAGE-enhancer (17 %)). *Cis* eQTL associations are presented in Additional file 5: Table S14.

#### Cis eQTL replication

To assess the reproducibility of the eQTLs identified in this study, we compared our results with previously published datasets. We used data from four brain eQTL studies, two large studies on blood-derived cell lines, and GTEx brain eQTL data [8, 9, 12–14, 42]. In total we could replicate 49,731 unique eQTL variants influencing 1139 CAGE-clusters or 47 % (32 % replicated from brain microarray studies, 23 % from blood-derived cell lines microarray studies, 36 % in all included microarray studies, and 35 % in the three GTEx RNA-seq datasets). The highest replication rates were identified from the more recent RNA-seq eQTL data, specifically from the brain cortex dataset (33 %). Variant-gene ID combination replication was performed in the three GTEx RNA-seq eQTL datasets and this resulted in a replication rate of 23.2 % (558 unique gene IDs). Some eQTL signals were replicated in almost all investigated eQTL datasets like the eQTL influencing the expression of *C2orf74* and *XRRA1*. Overall, these replication rates are in line with those found in previous studies, in particular considering that some of the eQTL types previously identified (including splicing eQTLs) cannot be detected using CAGEseq [9, 43]. Alternative explanations for the non-replicated eQTL signals could be genomic differences that are cohort-specific and the inclusion in our study of variants derived from exome sequencing and CAGEseq. It is also important to note that eQTL signals are not always consistent between brain regions and thus extrapolation to other regions or tissues should be looked at with caution [9].

We then performed indirect cis-eQTL validation by replicating known eQTL characteristics such as the enrichment of eQTLs near TSS, in variants according to their genetic location (e.g. UTRs), and in variants in close proximity of functional elements (e.g. enhancers) [9, 44]. We used all included variants as a measure for genome average and compared this with the location of sentinel variants, observing a decrease in intergenic variants and enrichment in all other genomic locations in the sentinel variants (Additional file 2: Table S10). In addition, we searched for overlap in genomic locations with two distinct types of data that mark regulatory elements: H3K27ac ChIP-seq and DHS data. Again, we used all included variants as a measure for genome average. Here, we observed a clear enrichment for functional elements in sentinel variants (Additional file 2: Table S11). Furthermore, we investigated the distance between the CAGE-clusters and the associated variants for both coding and non-coding eQTLs. Although we detected eQTLs as far away as 1 Mb from the TSS for both coding and non-coding transcripts, 82 % of the associated sentinel variants are located within 200 kb and 65 % are located only 50 kb from the influenced CAGE-cluster. Thus, we confirm previous findings that the strength of the eQTLs is inversely correlated with its distance to the TSS (Additional file 2: Figure S7) [14].

#### Trans eQTL discovery and replication

*Trans* eQTL analysis led to the identification of 7028 variants influencing 523 unique CAGE-clusters. Of these, 55.1 % (288) consisted of coding CAGE-clusters while 16.8 % (88) corresponded to non-coding CAGE-clusters, 10.3 % (54) were intergenic, and 17.8 % (93) mapped to retained introns, nonsense mediated decay, or other RNA biotypes (Additional file 6: Table S15). As previously described, *trans* eQTL signals generally display low replication rates across studies [43]. We identified nine variants from previous *trans* eQTL datasets that were also present in our *trans* eQTL results, eight from Gibbs et al. [12] and one from Byrois et al. [8]. However, all influenced CAGE-clusters were located on a different chromosome or in a different part of the chromosome with respect to the eQTLs previously reported. Despite using tissue-matched datasets in our analysis we could not replicate any of the identified trans-eQTLs; therefore, we did not consider them for further analysis. *Trans* eQTL associations are presented in Additional file 6: Table S15.

### eQTLs are enriched for non-coding genes

**Fig. 2:**
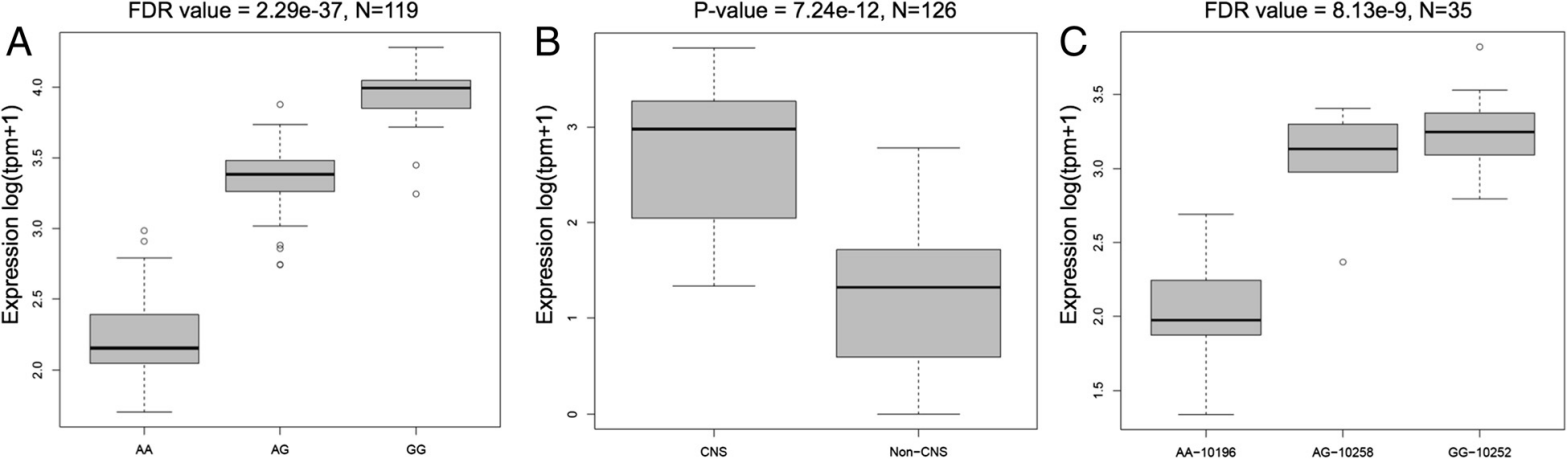
Association between rs320881 and the expression of *AC012309*.5 (*LINC01535*). **a** *Boxplot* showing the association between rs320881 and the expression (log(tpm + 1)) of *AC012309*.5 (*LINC01535*) with a FDR of <2.29E-37. Individuals carrying the non-reference allele (G) have a higher expression compared to reference allele (a) carriers. **b** *Boxplot* showing that *LINC01535* is predominantly expressed in central nervous system (CNS) tissues (n = 126) as compared to non-CNS tissues based on FANTOM5 expression data (*p* value 7.24E-12, Mann–Whitney U test one-sided). **c** *Boxplot* showing expression differences in *LINC01535* in three of the donors used in the FANTOM5 project to generate CNS tissues expression data. The three donors show different *LINC01535* expression patterns according to their rs320881 genotypes (FDR value of <8.13-09)

In recent years, non-coding RNAs have generated great interest, especially in brain research [45]. However, only few non-coding RNAs have been fully functionally characterized (see, for example, the role of *XIST*, *HOTAIR*, and Uchl1 antisense in X chromosome inactivation, epigenetic regulation, and gene regulation, respectively [46–48]). There is accumulating evidence that non-coding RNAs play a pivotal role in brain development and brain-related diseases (see Qureshi et al. for review [45]). In addition, it has been shown that brain expresses more non-coding RNAs as compared to other tissues [49, 50]. Taken together, it is interesting to identify the non-coding transcripts that are influenced by DNA variants and indirectly could alter the expression of coding transcripts. Here we identified 509 eQTLs that correlate with the expression of non-coding transcripts (processed transcripts, antisense transcripts, and lincRNA), 21 % of the total identified eQTLs. We found that expression differences for lincRNA *AC012309.5* (*LINC01535*) are correlated with variant rs320881 (Fig. 2a). *LINC01535* is located on chromosome 19q13.12 between two zinc finger proteins, *ZNF383* and *HKR1*, both involved in transcriptional regulation of the mitogen activated protein kinase (MAPK) signaling pathway, which activates transcription factors related to learning, memory, cell proliferation, and apoptosis [51, 52]. Suggestive evidence for linkage has been reported for schizoaffective disorders to the chromosomal location of these genes and abnormal activity of the MAPK signaling pathway has been observed in frontal cortical areas on postmortem brains in schizophrenia patients [53, 54]. Within the FANTOM5 expression data, we observed that *LINC01535* is predominantly expressed in CNS tissues (Fig. 2b) and expression differences between different FANTOM5 brain donors can be explained by the genotypes for rs320881 similar to the findings of our current study (Fig. 2c). It is tempting to speculate that *LINC01535* plays a role in MAPK signaling and it would be of interest to study if, for example, *LINC01535* plays a role in the (transcriptional) regulation of *ZNF383* and *HKR1* and thereby explain the identified association with schizophrenia.

Recently, it has been shown that lincRNAs are more prone to be contained within eQTLs as compared to protein-coding genes [55]. We replicated this finding for all non-coding transcripts (antisense and processed transcripts and lincRNAs) and identified a significantly higher fraction of eQTLs for non-coding CAGE-clusters as compared to coding CAGE-clusters (*p* value <5.73E-27, Chi-square = 105.6). Furthermore, when non-coding transcripts were identified as an eQTL, their FDR value was significantly lower than for coding transcripts (2.01E-11 Mann–Whitney U test one-sided). The largest difference was found between coding transcripts and processed transcripts (3.24E-8 Mann–Whitney U test one-sided), followed by coding transcripts versus lincRNAs or antisense (3.84E-4 and 4.67E-4, respectively, Mann–Whitney U test one-sided). No statistically significant differences were found between the different types of non-coding transcripts (Mann–Whitney U test two-sided). Additionally, the genomic distance between the CAGE-cluster and the sentinel variant of non-coding eQTLs was significantly smaller than for coding eQTLs (*p* value <9.13E-5 Mann–Whitney U test one-sided). This effect was strongest between coding transcripts and lincRNAs (*p* value <4.53E-4 Mann–Whitney U test one-sided), followed by antisense and processed transcripts (*p* value <6.34E-3 and 0.056 Mann–Whitney U test one-sided, respectively). No statistical difference was found within the non-coding group.

### Identifying “causal” variants for eQTLs

Variants obtained from CAGEseq data are of special interest as the likelihood that they represent the actual variant causing the expression changes is much higher compared to tagging SNPs used on microarrays. This is because they are located in the very close vicinity (<30 bp) of the actual TSS and thus likely represent variants that directly influence the binding strength of the transcription complex. Variants obtained from exome sequencing are of special interest as well as they could be close to the TSS as well or could influence transcript stability. Indeed, we find in our data that CAGEseq variants were 85 times more often identified as a sentinel eQTL compared to BeadChip and imputed variants and exome sequencing variants showed a similar, albeit weaker, trend (77 times, respectively).

We then used RegulomeDB to predict a causality score for all the sentinel variants. We obtained causality scores for all 2376 unique sentinel variants, with 127 scoring 1 (likely to affect binding and linked to expression of a gene target) and 188 scoring 2 (likely to affect binding), suggesting that at least 13 % of all our sentinel variants are likely to be the causal variant. When focusing on sentinel variants within 1 kb of the TSS, we observed 31 % of all variants scoring 1 or 2. Besides, CAGEseq variant eQTLs had a lower average RegulomeDB score compared to non CAGEseq variant eQTLs (3.1 vs 4.9) supporting the more likely regulatory role of CAGEseq variants. The complete list of the RegulomeDB scores is provided in Additional file 7: Table S16.

### Using eQTLs to interpret GWAS results

As the majority of the identified variants from GWAS are located in non-coding regions, the interpretation of their consequences on a molecular level remains difficult. A straightforward method to interpret the biological effect underlying the risk loci is to correlate the GWAS loci with eQTLs [56]. We therefore intersected our list of eQTL variants with the GWAS variant catalog [1]. Overall, we obtained an overlap with 381 variants identified in 557 separate GWAS, representing 253 individual eQTLs signals from the current study. Of these 557 GWAS associations, 359 reached whole genome significance in their GWAS (*p* value <8e-5, Additional file 8: Table S17).

Using CAGEseq as an expression profiling technique allows the identification of eQTLs for specific TSSs of transcripts. Therefore, we can correlate variants in the identified risk loci with the individual transcripts of a gene instead of measuring the effect on all gene transcripts combined, which is important for designing follow-up studies. This is exemplified by the inflammatory bowel disease (IBD) locus on chromosome 2q25 associated with the rs2382817. Fifteen genes are present in the region of association including the *PNKD* and *TMBIM1* genes, in which the variant occurs. We identified an eQTL for a single TSS, downstream of the primary TSS of *TMBIM1* (TMBIM1_tss2), for two transcripts variants of *TMBIM1* (Ensembl transcripts ENST00000418569 and ENST00000444000) encoding protein isoforms lacking five of the seven transmembrane domains. *TMBIM1*, a member of the transmembrane Bax Inhibitor-1 containing motif proteins family, is located mainly in the Golgi apparatus and in the endoplasmatic reticulum (ER) [57, 58]. It controls ER-Ca2+ homeostasis and dynamics through a complex network of interactions, likely pH-dependent, involving amino acids residues located in the carboxyl terminal cytosolic region, conserved among all the protein family members and missing in the ENST00000418569 and ENST00000444000 transcripts. It has been suggested that the *TMBIM* proteins exert anti-apoptotic activities likely related to their capacity of controlling CA2+ flux at the ER and Golgi [59]. Intestinal epithelial cells apoptosis contributes to the development of IBD [60]. It is therefore tempting to speculate that change in expression of ENST00000418569 and ENST00000444000 transcripts might lead to reduced anti-apoptotic activity and therefore increase the risk to IBD.

A similar scenario can be found for rs3744028 and the rs1055129, which have been consistently associated with white matter hyperintensity [61–63]. The two variants are physically close and influence a CAGE-cluster located 500 bp downstream of the main TSS for two isoforms of *TRIM47* (Ensembl transcripts nonsense mediated decay ENST00000587339 and retained intron ENST00000587774) rather than the annotated main TSS of *TRIM47*. There is good evidence that intron retention (IR), once recognized merely as a consequence of mis-splicing leading to failed excision of intronic sequences from pre-messenger RNAs, is part of a physiological mechanism of gene expression control [64]. In particular, it has been shown that the level of intron retention in genes involved in differentiation processes increases markedly during subsequent stages of maturation, resulting in greatly reduced protein levels due to nonsense mediated decay. IR provides therefore an energetically favorable level of gene expression control important to sustained gene translation. It is possible such a mechanism is more widespread and therefore a change in expression of the ENST00000587774 transcript might play a role in regulating *TRIM47*.

Because the presented eQTL dataset was derived from brain material, we focused mainly on GWAS for brain-related disorders (which we defined as traits influenced by mental state or known brain diseases). We identified 58 variants (16 reached whole genome significance in the respective study) and identified eQTLs for genes at risk loci for migraine, multiple sclerosis, PD, Alzheimer’s disease (AD), and schizophrenia (Additional file 8: Table S17).

### Parkinson’s and Alzheimer’s disease GWAS loci eQTLs

We identified four CAGE-clusters influenced by an eQTL for the *MAPT* locus on chromosome 17q21. Two divergent *MAPT* haplotypes, H1 and H2, have been described with distinct linkage disequilibrium patterns across a 1.08–1.49 Mb region reflecting the presence of a common inversion. The H1 haplotype has been associated with progressive supranuclear palsy, corticobasal degeneration, PD, and AD [65, 66], while the H2 haplotype has been linked to recurrent deletion events of *KANSL1*, a gene that encodes a nuclear protein that plays a role in chromatin modification associated with the 17q21.31 microdeletion syndrome, a disease characterized by developmental delay and learning disability [67, 68]. In addition, recurrent partial duplications of *KANSL1* have been reported on both haplotypes [69, 70]. An eQTL for *MAPT* has been reported, [14, 71, 72] but other studies could not confirm this finding and instead found an eQTL associated with the alternative splicing of exon 3 of *MAPT* [73–75]. We did not replicate the eQTL for *MAPT* in our data because we solely focus on expression differences in the TSS, but we detected eQTLs in this region influencing two CAGE-clusters of *KANSL1*, one for a CAGE-cluster of *KANSL1-AS* (*LOC644246*) and a CAGE-cluster of *CRHR1*. The eQTLs containing *CRHR1*, *KANSL1*, and *KANSL1-AS* have previously been identified [9, 76]. Interestingly the eQTL for *KANSL1* was discussed in a recent meta-analysis for AD using more than 17,000 cases where a new genome-wide significant association was identified for rs2732703 on chromosome 17q21.31 approximately 200 kb downstream of *MAPT* in *APOE4* negative cases [66]. By conditioning the analysis on *MAPT* haplotypes, the authors found that the causal variant(s) are more likely located in a DNA segment between the 5′ end of *KANSL1* and 5′ end of *LRRC37A* and not within *MAPT* or another gene distal to *LRRC37A*. In the four eQTLs identified in our study, we detected higher expression for the H2 haplotype, which is associated with a reduced risk for AD [66]. Duplications and partial duplications of *KANSL1* occur on both the H1 and H2 haplotypes (and subtypes) [69, 70] and with the use of genotype and CAGEseq expression data we cannot determine the exact mechanism behind these eQTLs, but our data and the recent meta-analysis for AD suggest a role for disease risk of *KANSL1* or *KANSL1-AS* [66]. Future studies that allow the full reconstruction of genomic variants and duplication events in individuals combined with full transcript expression and epigenetic data can hopefully resolve this.

**Fig. 3:**
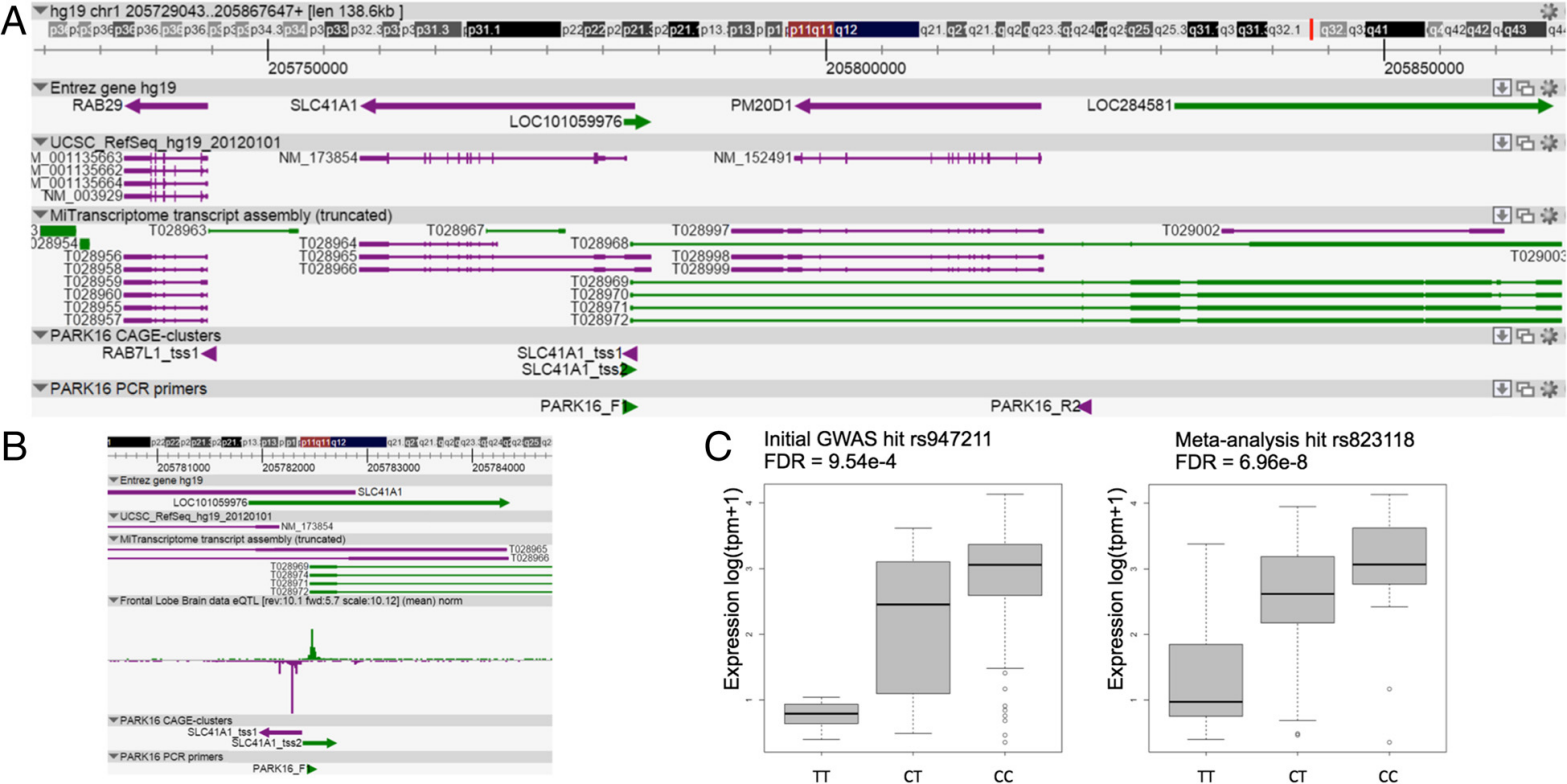
The PARK16 locus harbors an eQTL that overlaps with a PD GWAS loci for a new gene/transcript. **a** Overview of the PARK16 locus where several genes are present (Entrez Gene hg19) and several transcript predictions from MiTranscriptome. We identified an eQTL for an antisense CAGE-cluster to the *SLC41A1*gene (SLC41A1_tss2), for this CAGE-cluster multiple transcript predictions are present in the MiTranscriptome database. **b** Zooming in on the actual transcription start site of *SLC41A1* gene shows CAGEseq expression of the *SLC41A1*gene (SLC41A1_tss1, *purple*) and the CAGE-cluster (SLC41A1_tss2, *green*), matching the MiTranscriptome predictions. **c** *Boxplots* showing the eQTL association of the two PD GWAS associated variants, rs947211 and rs823118, with the expression (log(tpm + 1)) of antisense transcript to the *SLC41A1*gene, all showing an additive effect

Another example of an overlapping PD GWAS locus and our eQTLs is the PARK16 locus, which is associated with sporadic PD for SNPs rs947211 and rs823118, respectively [4, 77, 78]. The PARK16 locus is located on chromosome 1q32 and contains four genes, of which *RAB7L1* (*RAB29*) and *SLC41A1* have been proposed as possible causal genes (Fig. 3a) [79–82]. The Na+/Mg2+ exchanger *SLC41A* is a key component of cellular magnesium homeostasis and *RAB7L1*, together with *LRRK2* assures proper functioning of the retromer complex that links the endolysosomal protein degradation system with the Golgi apparatus. We found that both GWAS SNPs influence the expression of an antisense CAGE-cluster (SLC41A1_tss2), which is part of a bidirectional promoter of *SLC41A1* (Fig. 3b), where having the reported risk allele (T) results in higher expression of SLC41A1_tss2 (Fig. 3c). SLC41A1_tss2 would function as TSS for *LOC101059976*, however the NCBI record of this gene was withdrawn because it was not predicted in a later annotation*.* Analysis of MiTranscriptome data suggests that SLC41A1_tss2 is the TSS for the gene *LOC284581*, which would be a gene/transcript spanning *PM20D1* on the opposite strand of *RAB7L1*, *SLC41A1*, and *PM20D1* (Fig. 3a) [40]. Partial validation of this eQTL was found by an exon probe eQTL of variant rs1772143 to influence *LOC284581* expression [9]. To test this prediction, we performed a PCR on cDNA from brain using sets of primers covering SLC41A1_tss2 and the first exon predicted from the MiTranscriptome RNA-seq data (Fig. 3a and 3b). Sanger sequence data from the PCR supported the prediction of MiTranscriptome, suggesting that SLC41A1_tss2 is a TSS for *LOC284581* (Additional file 2: Figures S8–S10). It is tempting to speculate that this novel gene could play a role in the risk for developing PD at the PARK16 locus by, for example, transcriptional regulation, RNA stability, or alternative splicing of neighboring genes including *SLC45A3*, *NUCKS1*, *RAB7L1*, *SLC41A1*, and *PM20D1*. We found no clear correlation between expression levels, but our data only allow for measuring expression at the transcription start and other methods are needed to study changes in RNA stability, alternative splicing, or changes on protein level. Overall, more research is needed to find the function of this “new” gene/transcript and whether it is involved in PD.

### Schizophrenia NRGN GWAS loci eQTL

*NGRN* is an important risk factor for schizophrenia. Association studies have identified common variants in this gene region that are associated with an increased risk for schizophrenia [83]. In particular, rs12807809 genotypes have been correlated to specific neuropsychological symptoms in schizophrenia and brain structure [84, 85]. While several eQTLs have been described for schizophrenia loci in brain, no eQTLs have been yet identified for the *NRGN* locus [86]. Previous attempts to explain the effect of GWAS variant rs12807809 in the locus by eQTL analysis have been inconclusive [87]. Ohi and colleagues, however, observed a diplotype for SNPs rs12807809 and rs12278912 that increases the risk for schizophrenia and influences *NRGN* expression in immortalized lymphoblasts [88], and it was suggested that it has an effect on the intelligence quotient of schizophrenia patients [89].

**Fig. 4:**
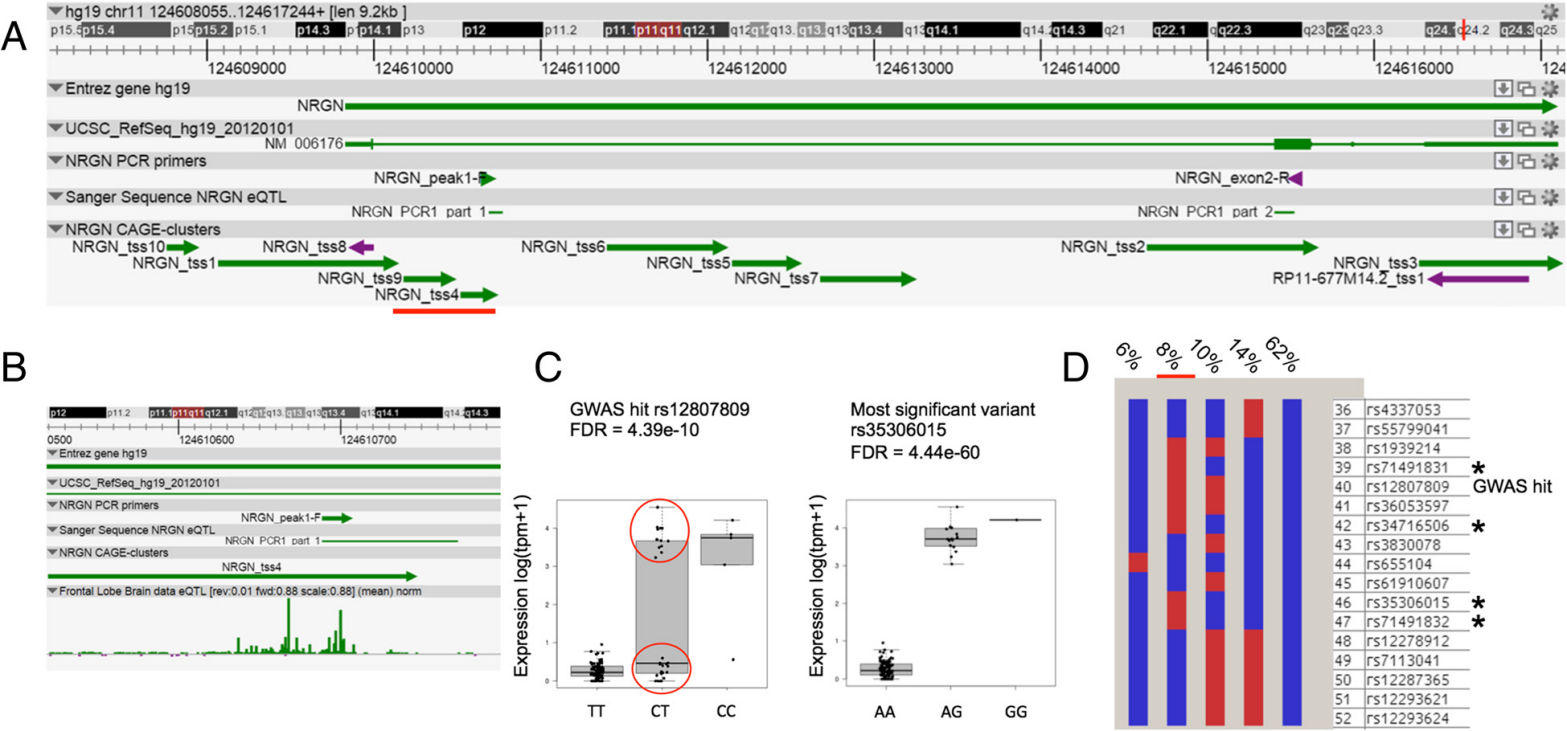
An intragenic transcript of *NRGN* is influenced by a schizophrenia GWAS eQTL. **a** *NRGN* region overview with all the TSSs identified by CAGEseq. Here we identified 11 CAGE-clusters for the *NRGN* locus, nine sense and two antisense. *Underlined in red* is the NRGN_tss4 influenced by rs12807809, the highest associated SNP from the schizophrenia GWAS study Stefansson et al. [83]. **b** Zooming in on the actual transcription start site of NRGN_tss4 shows CAGEseq expression signal. Primers were designed based on the CAGEseq expression and on the first exon this resulted in part of a novel transcript in this *NRGN* locus. **c** Two *boxplots* showing the association of GWAS hit SNP rs12807809 and the SNP with the highest *p* value rs35306015 with the expression (log(tpm + 1)) of NRGN_tss4, respectively. For the GWAS hit rs12807809, the heterozygous carriers are clearly separated in two groups with 19 individuals showing the same expression pattern as the homozygotes AA while the remaining 13 carriers show the expression pattern of the homozygotes BB suggesting an underlying effect. This effect is not present for the rs35306015 variant. The minor allele frequency (MAF) for rs35306015 in our cohort (MAF = 0.076) is similar to the European frequency in 1000 Genomes. **d** Haplotypes drawn with Haploview using genotypes from all the 119 included individuals in this study. Note the specific haplotype with 8 % frequency for the 17 individuals presenting highest expression for NRGN_tss4, highlighted with a *red bar*. Of the 17 haplotype carriers, 16 are heterozygous and one homozygous. *Black asterisks* represent variants unique to this haplotype and in perfect linkage disequilibrium in our dataset

We identified 11 CAGE-clusters for the *NRGN* locus, nine sense and two antisense (Fig. 4a). Intragenic CAGE-clusters are a common feature for this gene based on our and FANTOM5 data and are also identified in the mouse ortholog *Nrgn* [6, 90]. Additionally, functional characterization of intragenic *Nrgn* transcripts have already been described using (fluorescent) in situ hybridization and it is proposed that the differential regulation of sense and antisense transcripts will increase the diversity of post-transcriptional regulation [90].

We identified several eQTLs for the *NRGN* locus. One of the CAGE-clusters, NRGN_tss4, which is 500 bp downstream of the main TSS (Fig. 4a and 4b), correlates with the highest associated SNP from a recent GWAS, rs12807809 [83]. Homozygous individuals for the reference allele have a lower expression of NRGN_tss4 in comparison to homozygous for the alternative allele. Interestingly, heterozygous carriers show a clear separation into two groups, one behaving as the homozygous reference and the other as the homozygous alternative allele carriers (Fig. 4c), suggesting an additional factor present, for example, a haplotype effect. In order to investigate this, we estimated haplotypes based on the available genotype data and identified a specific haplotype spanning 17 variants surrounding rs12807809 for the 17 individuals that have a high expression of the CAGE-cluster (Fig. 4d). Four variants (all non-reference alleles) were in perfect linkage disequilibrium and strongly correlated to the expression of NRGN_tss4: rs71491832, rs35306015, rs71491831, and rs34716506 in a 6.2 kb region (Fig. 4b). We replicated these findings by performing PCRs on cDNAs from six additional donors using sets of primers in the NGRN_tss4 and *NRGN* exon2 (Fig. 4a and 4b). The results confirm that the expression of this CAGE-cluster is genuine and that expression is only detectable in the individuals carrying the haplotype described here (Additional file 2: Figures S11–S13). It is important to notice that NRGN_tss4 and rs35306015 are in close proximity of predicted functional elements (predicted enhancer and CTCF site [7]), which could explain this eQTL effect.

Spearman correlation analysis performed on all the *NRGN* CAGE-clusters showed a high correlation (>0.64) between the expression of the main TSS (NRGN_tss1) and most of the intragenic CAGE-clusters with the exception of NRGN_tss4 and NRGN_tss9 (due to the eQTL effect described) and NRGN_tss8 (most likely because of low expression; Additional file 2: Figure S14). When using only the 17 samples with expression of NRGN_tss4 a high correlation (>0.65) was also present with the main TSS. Considering these data, it is likely that alterations in expression of human *NRGN* intragenic TSSs could results in a different post-transcriptional regulation of human *NRGN* gene. This has similarities to the mouse locus for which several sense and antisense transcripts have been reported whose expression is spatiotemporally regulated from development until the adult mouse [90]. This complex expression pattern in both human and mouse is consistent with the role of *NGRN* in synaptic long-term potentiation, which requires a precise and highly dynamic regulation of gene expression in response to external stimuli. The functional role of the eQTL therefore warrants further investigations to confirm the transcriptional regulation of the *NRGN* gene and the potential role on brain development and schizophrenia.

## Conclusions

One of the main hurdles to translate findings from GWAS studies into biology is that the vast majority of GWAS risk loci are located in non-coding or poorly annotated regions making the interpretation of their role in disease etiology challenging. eQTL analysis has emerged as an important tool to help understanding the molecular consequences of human variation but has mostly focused on genotypes from microarrays containing tagging SNPs and expression data have been mostly generated for protein-coding genes. By performing eQTL analysis on CAGEseq expression data obtained from a series of human postmortem frontal lobe samples, in combination with genome wide array based genotyping, exome sequencing, and variants derived from CAGEseq, we have generated a rich resource for researchers to mine. Overlapping eQTLs with GWAS loci made it possible to create new hypotheses for increased risk of disease via molecular effects, but further confirmation is needed with additional statistical tests and experimental follow-ups. Our data contain both coding and non-coding transcripts and has the added value that we have identified eQTLs for variants directly adjacent to TSS. We demonstrated that these have a high likelihood of being causal variants, which will be an important tool to understand what the molecular mechanisms underlying genetic risk loci are.

## Abbreviations

AD, Alzheimer’s disease; BWA, Burrows-Wheeler Aligner; CAGE, Cap analysis gene expression; CAGEseq, Cap analysis gene expression sequencing; cDNA, complementary DNA; DHS, DNase hypersensitivity sites; eQTL, Expression quantitative trait loci; ER, Endoplasmatic reticulum; FDR, False discovery rate; GATK, Genome Analysis Toolkit; GWAS, Genome wide association studies; HWE, Hardy-Weinberg equilibrium; IBD, Inflammatory bowel disease; IR, Intron retention; MAF, Minor allele frequency; MAPK, Mitogen activated protein kinase; MDS, Multidimensional scaling; NABEC, North American Brain Expression Consortium; PCA, Principal component analysis; PD, Parkinson’s disease; PMI, Postmortem interval; RIN, RNA integrity number; RNA-seq, RNA sequencing; SNP, Single nucleotide polymorphism; tpm, Tags per million; TSS, Transcription start sites; UTR, Untranslated regions

## Additional files

Additional file 1: Table S1. All included samples with their characteristics. List of all the samples passing quality controls used in this study with sample details including gender, age, RNA integrity number, postmortem interval, number of mapped reads, included in exome sequencing, and brain bank origin. (XLSX 361 kb) Additional file 2: Supplementary data file containing supplementary figures S1 to S14 and supplementary Tables S2 to S11. (DOCX 2548 kb) Additional file 3: Table S12. List of all identified CAGE-clusters. List of all identified CAGE-clusters reported with their chromosomal locations gene ID, gene and transcript type class according to GENCODE version 17, and expression characteristics. (XLSX 2347 kb) Additional file 4: Table S13. Manual annotation of intergenic CAGE-clusters. Manual annotation of intergenic CAGE-clusters using the following public databases/datasets: RefSeq genes; FANTOM5 phase I permissive TSSs; Repetitive Elements database; CAGEseq expression derived enhancer dataset; frontal cortex H3K4me3 ChIP-Seq dataset. Overlap between datasets is provided. (XLSX 60 kb) Additional file 5: Table S14. All identified cis eQTL variants. List of all cis eQTL variants identified in this study including chromosomal position, gene ID, transcript class according to GENCODE, FDR values and Matrix eQTL statistics, SNPs characteristics, and distant between variants and CAGE-clusters. (XLSX 18351 kb) Additional file 6: Table S15. All identified trans eQTL variants. List of all trans eQTL identified in this study including details for CAGE-cluster and SNPs chromosomal positions, FDR values, and information about co-occurrence of trans and cis-eQTLs. (XLSX 1110 kb) Additional file 7: Table S16. All identified sentinel cis eQTLs. List of possible causal variant cis eQTL based on location and lowest *p* value including CAGE-cluster and SNPs chromosomal positions; gene ID; biotype class according to GENCODE; FDR values and RegulomeDB scores. (XLSX 419 kb) Additional file 8: Table S17. Overlap between GWAS database and identified eQTL variants. List of all the identified cis eQTL linked to GWAS variants. Provided in this table are: chromosomal locations of the CAGE-clusters and SNPs, FDR values, pubmed ID, and disease traits. (XLSX 207 kb)
