# Supplementary material for: Comprehensive promoter level expression quantitative trait loci analysis of the human frontal lobe
Source: Genome Med. 2016 Jun 10;8:65. doi: 10.1186/s13073-016-0320-1 (PMC4903003; doi:10.1186/s13073-016-0320-1)
Supplement: Additional file 2: — Supplementary data file containing supplementary figures S1 to S14 and supplementary Tables S2 to S11. (DOCX 2548 kb) [file 13073_2016_320_MOESM2_ESM.docx]

**Figure S1.** *Multidimensional scaling plot* of HapMap populations compared with the current studied population. The majority of our studied population (*green dots*) is tightly grouped with the CEU population (*black dots*, Europeans ancestry). However, one individual has some African ancestry (*blue dots*, Yoruba) positioned between the CEU and Yoruba populations. This individual was removed for subsequent analyses.

**
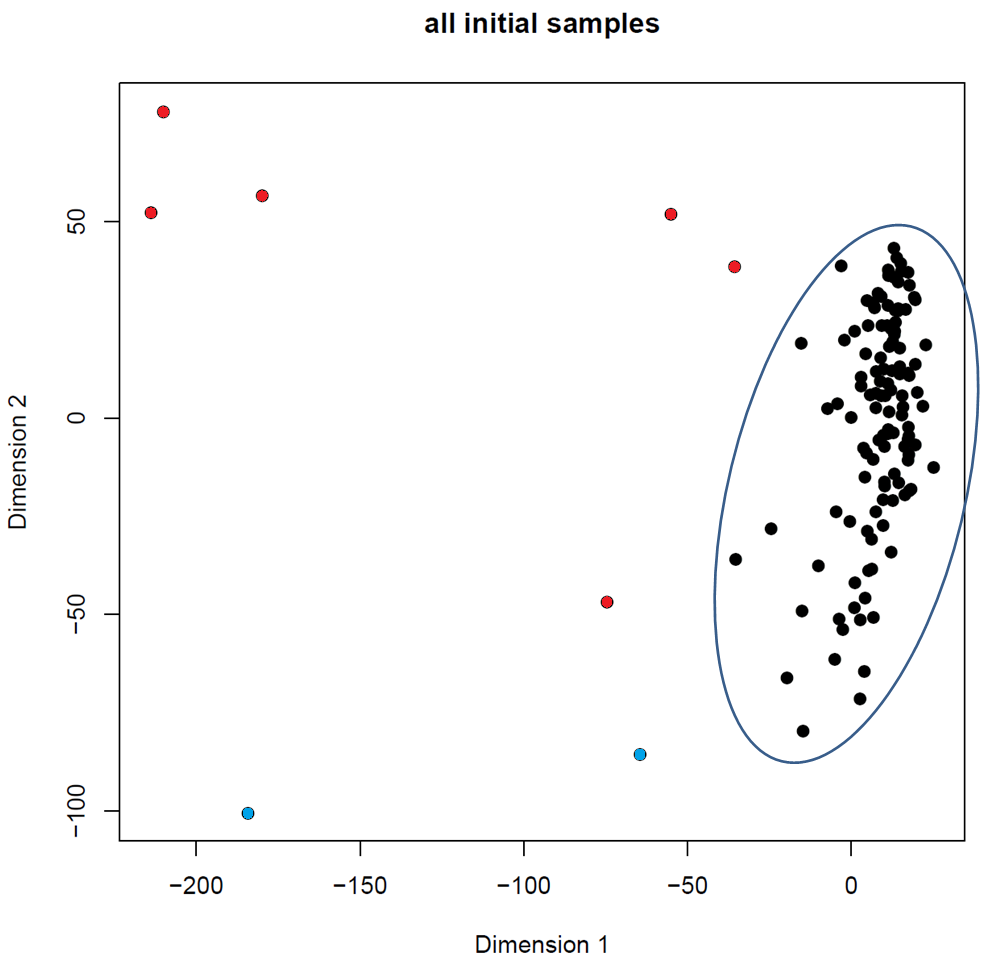
Figure S2.** *Multidimensional scaling plot* of CAGEseq expression data for all sequenced libraries. The *red dots* represent CAGEseq libraries with less than 1 million sequenced reads and the *blue dots* represent CAGEseq libraries with less than 25% of the reads mapping to the 5’UTR region of annotated genes. Since both numbers suggest failed or incorrect library preparation we removed these samples from downstream analyses. The samples included are encircled by the *blue ellipse*

**Figure S3.** *Multidimensional scaling plot* of CAGEseq data after outlier removal. No obvious substructure is visible in the data.

**
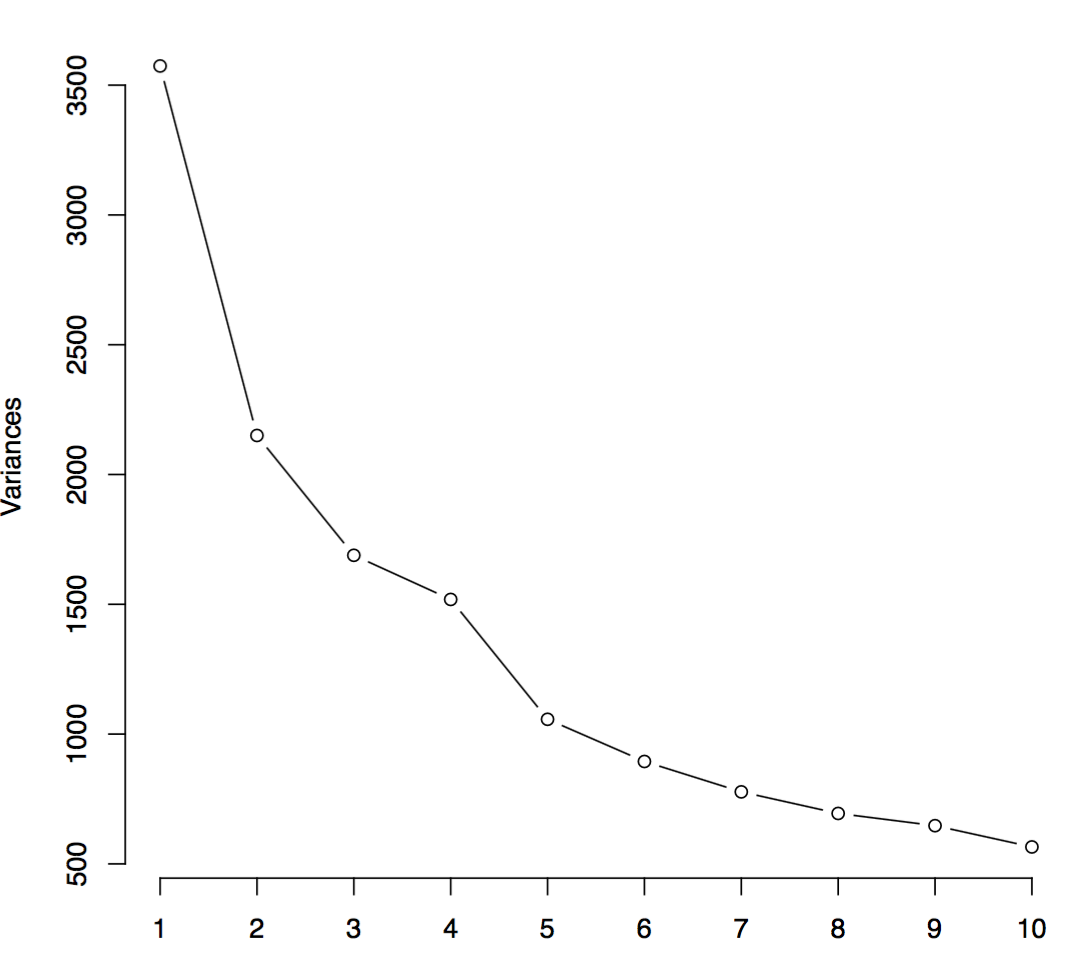
**

**Figure S4.** PCA of the CAGEseq expression data regressing out the four known including covariates (RIN, age, gender, and pmi). On the *y-axis* the variation is plotted and the *x-axis* is the dimensions.

**Figure S5.** Distribution of local linkage disequilibrium in all included variants and sentinel eQTLs. Local linkage disequilibrium (LD) was measured by the number of variants in a region of 1 MB upstream and downstream with an r2 >0.5. Two subsets of 100,000 variants were randomly chosen to represent all included variants and distribution was highly similar between all included variants and the sentinel eQTLs.

**Figure S6.** Distribution of minor allele frequency in all included variants and sentinel eQTLs. Minor allele frequency (MAF) was calculated for each variant and the distribution was plotted to identify potential confounding effect for enrichment analyses. All included variants appear to contain more variants with a lower MAF compared to sentinel variants.


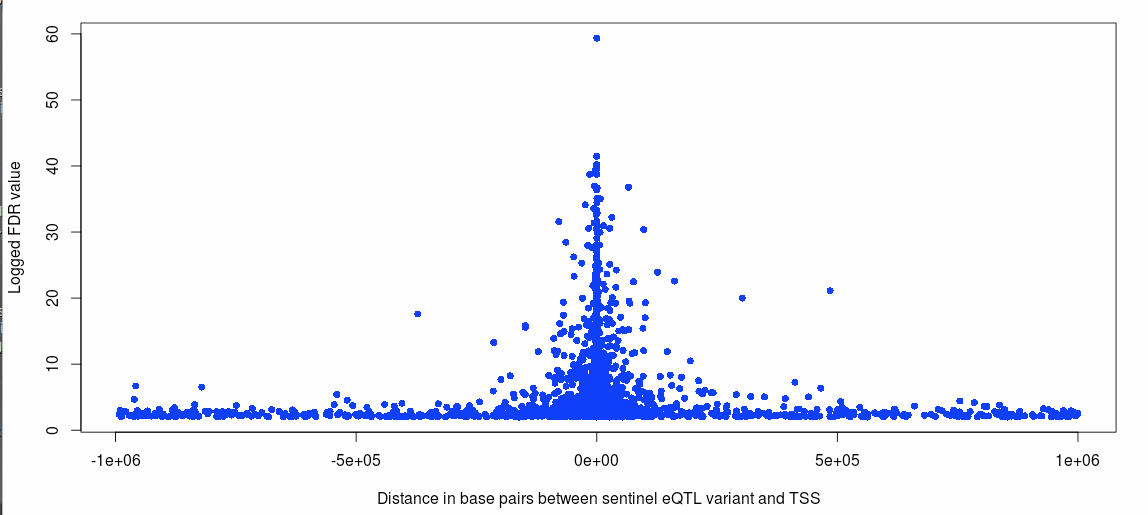


**Figure S7.** Sentinel eQTL variants are closer to the TSS when having a lower FDR-value. On the *x-axis* the distance in base pairs between the sentinel variant and the associated CAGE-cluster and on the *y-axis* the logged FDR value.


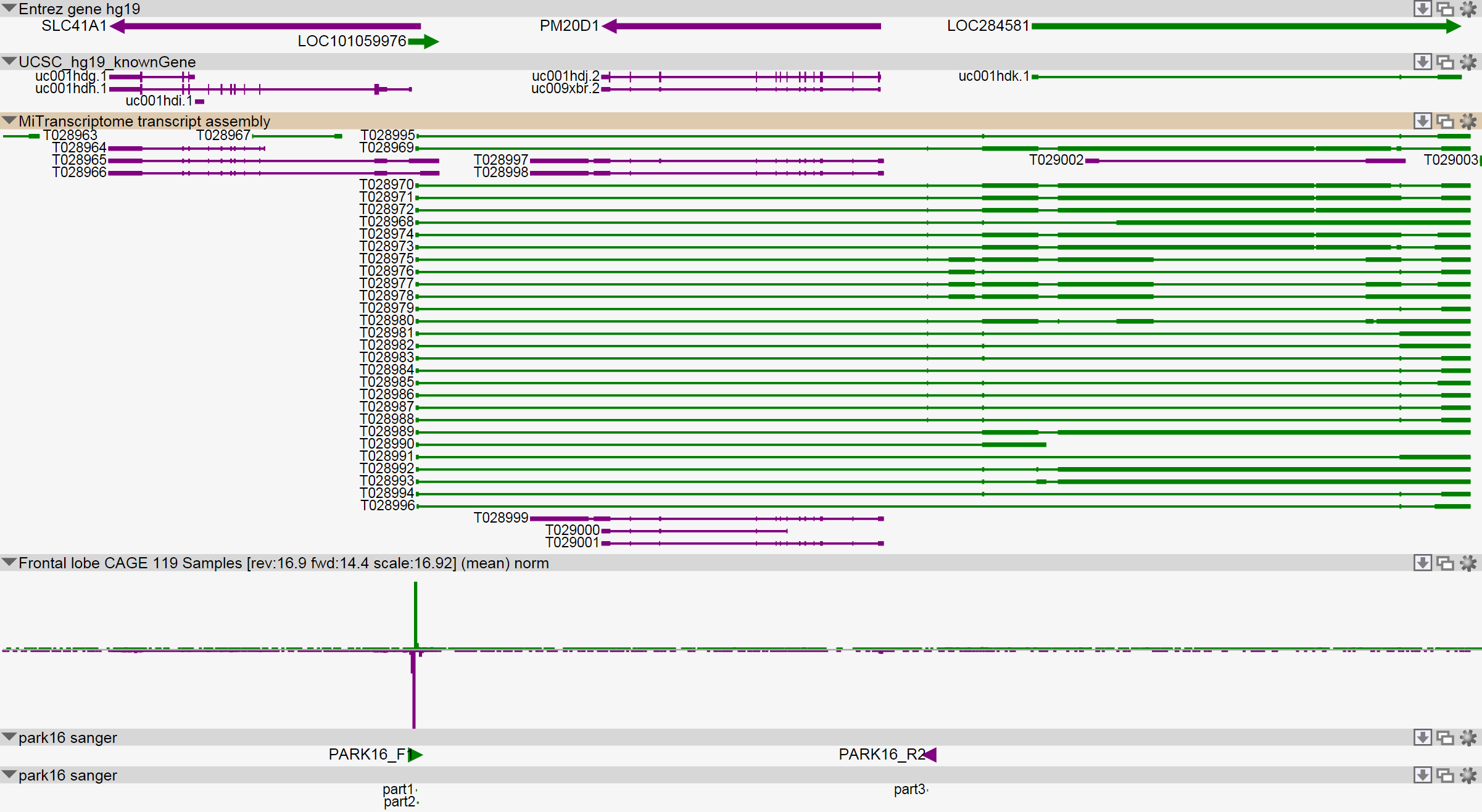
**Figure S8.** PARK16 locus overview and primer design. Two main expression CAGE-clusters are identified, one sense to *SLC41A1* and one antisense to *SLC41A1*. According to MiTranscriptome gene models, this antisense CAGE-clusters represents a transcript spanning the gene *PM20D1*. To validate this, primers were designed at the start site of the antisense and in the first exon of the MiTranscriptome gene models (Figure S7).

**
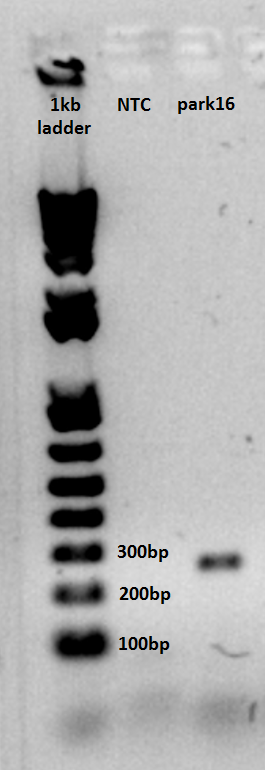
**

**Figure S9.** PARK16 new gene/transcript validation PCR product. Primers were designed based on our CAGEseq data (TSS) and on the MiTranscriptome data (first exon), see Table S12 for primers sequences (PARK16_F1 and PARK16_R1). A PCR product could be amplified by about 280 bp. Sanger sequencing results are presented in Figure S7.

**
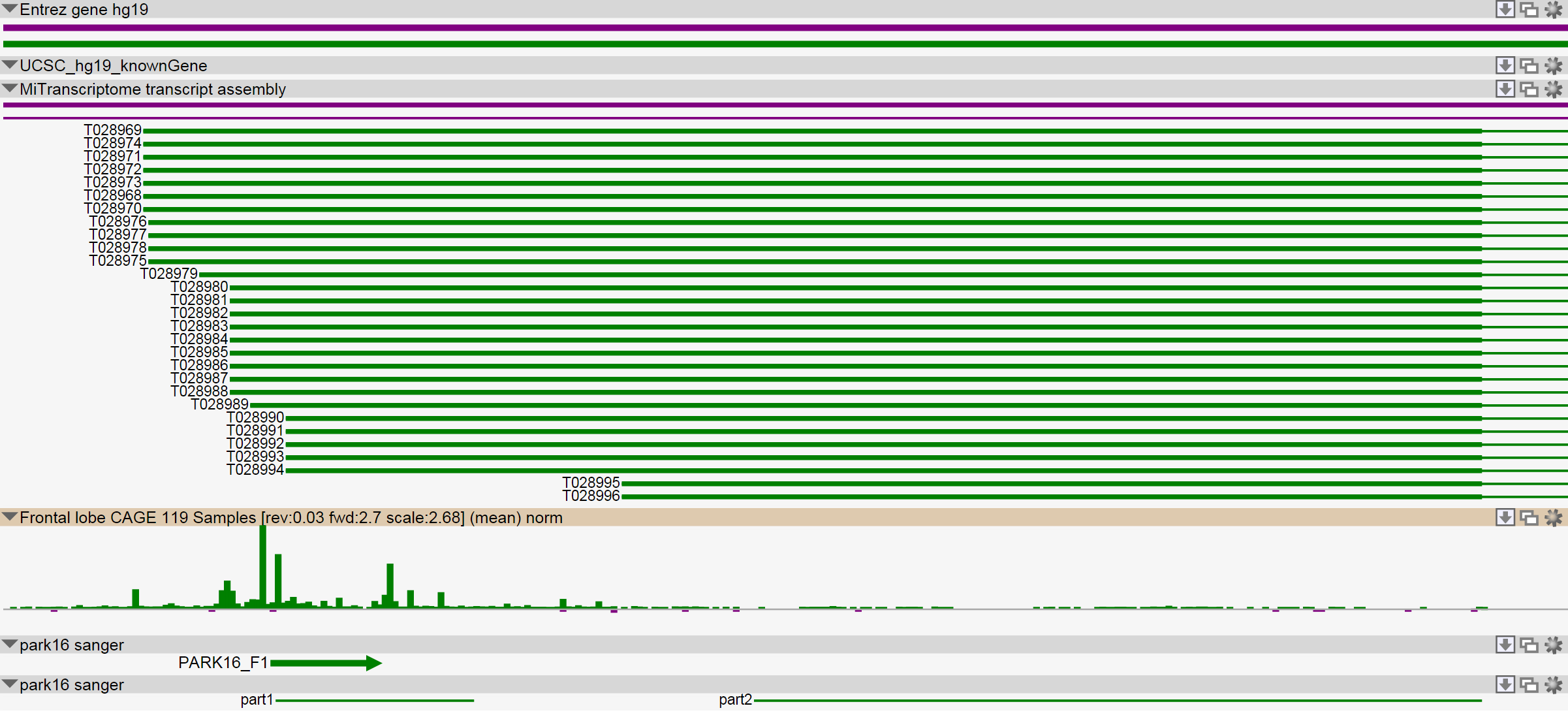
**

**B**

**A**

**
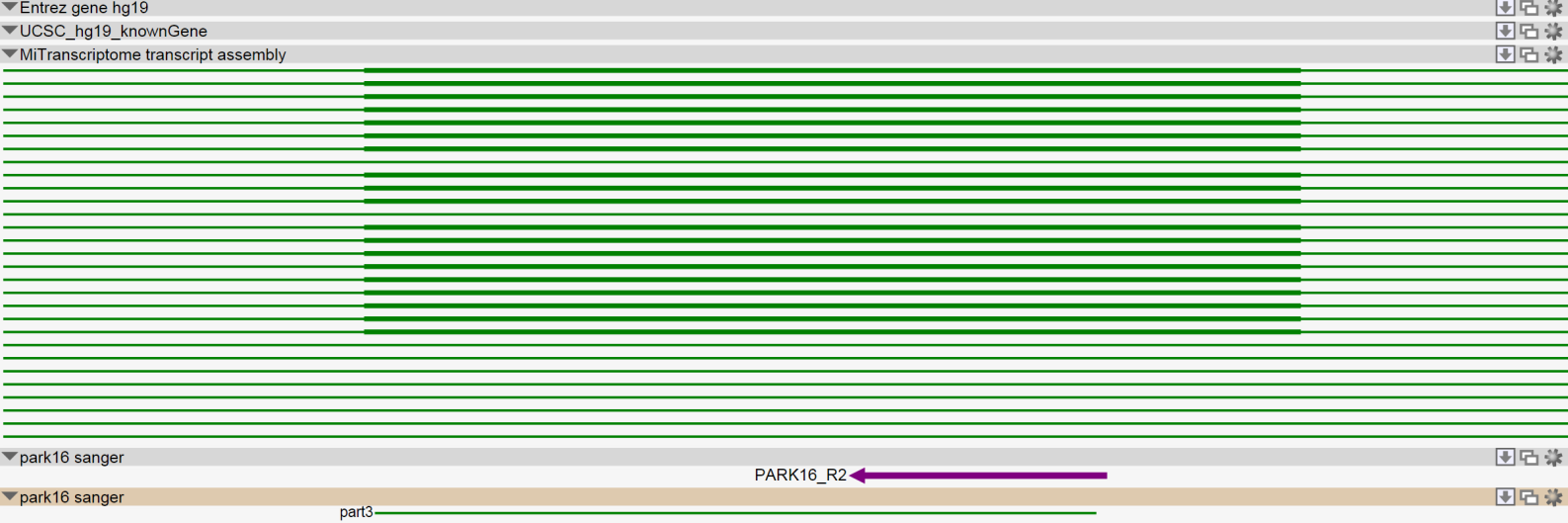
**

**Figure S10.** PARK16 new gene/transcript validation Sanger sequencing. **A** Zoomed in from Figure S5 focusing on the CAGE-cluster identified as eQTL. Using the primer directly located on the TSS we were able to validate the start site. **B** Zoomed in from Figure S5 focusing on the first exon of the MiTranscriptome gene model. Using the primer located on this exon we were able to validate the MiTranscriptome model and confirmed the exon boundaries of this transcript.


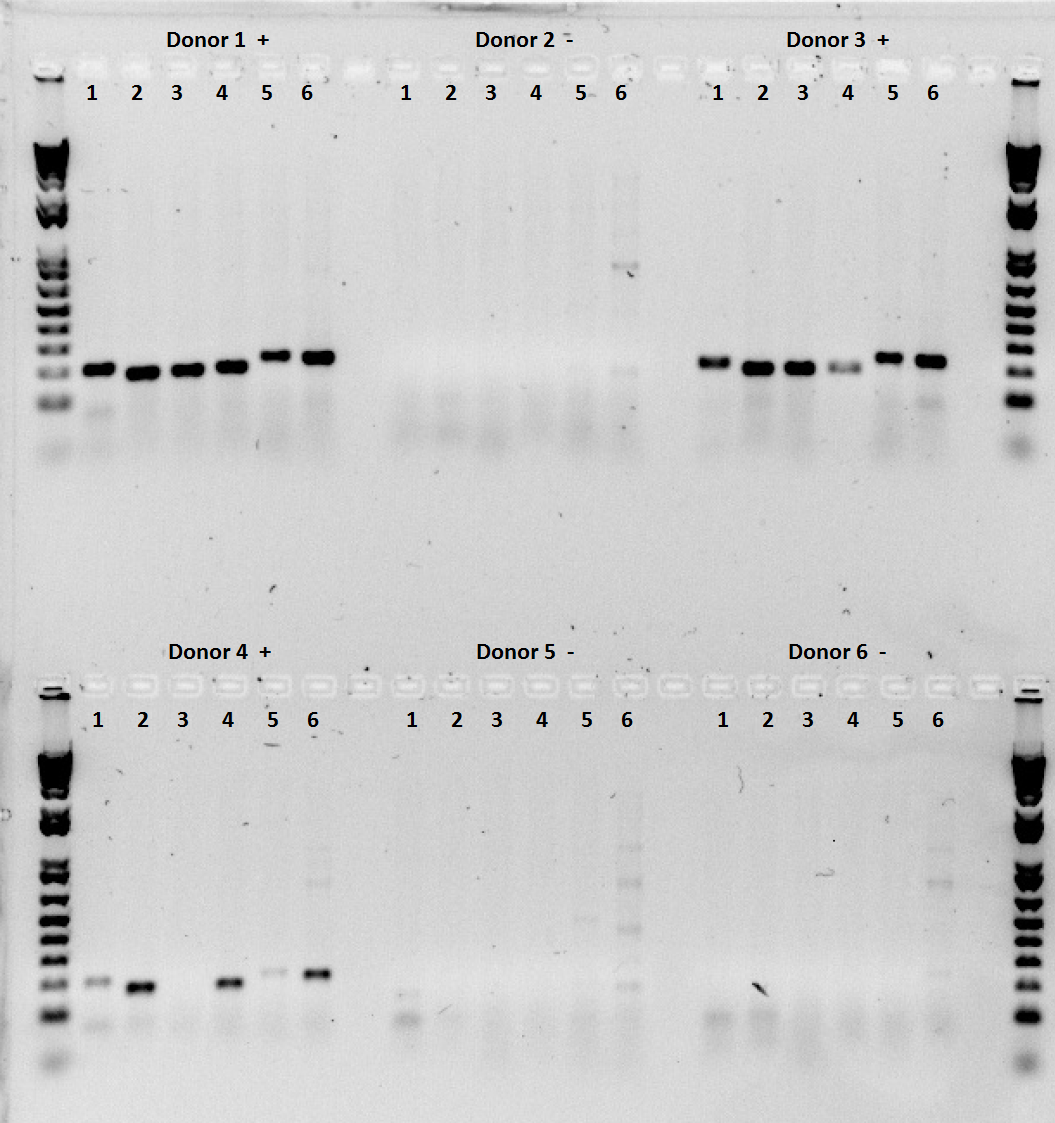


**Figure S11.** CAGE-cluster NRGN_tss4 eQTL experimental validation. *NRGN* eQTL validation, with donor number as in Table S11, showing amplification in the three samples which have expression CAGEseq signals. Primer set from Table S12 and primer combinations used as follows:

PCR1 = NRGN_peak1-F + NRGN_exon2-R (was Sanger sequencing from Donor 1)

PCR2 = NRGN_peak2-F + NRGN_exon2-R (was Sanger sequencing from Donor 1)

PCR3 = 3RACE_NRGN1-F + NRGN_exon2-R

PCR4 = 3RACE_NRGN2-F + NRGN_exon2-R

PCR5 = 3RACE_NRGN3-F + NRGN_exon2-R

PCR6 = 3RACE_NRGN4-F + NRGN_exon2-R

Genotyping confirmed that Donors 1, 3, and 4 carry the expression-specific haplotype.


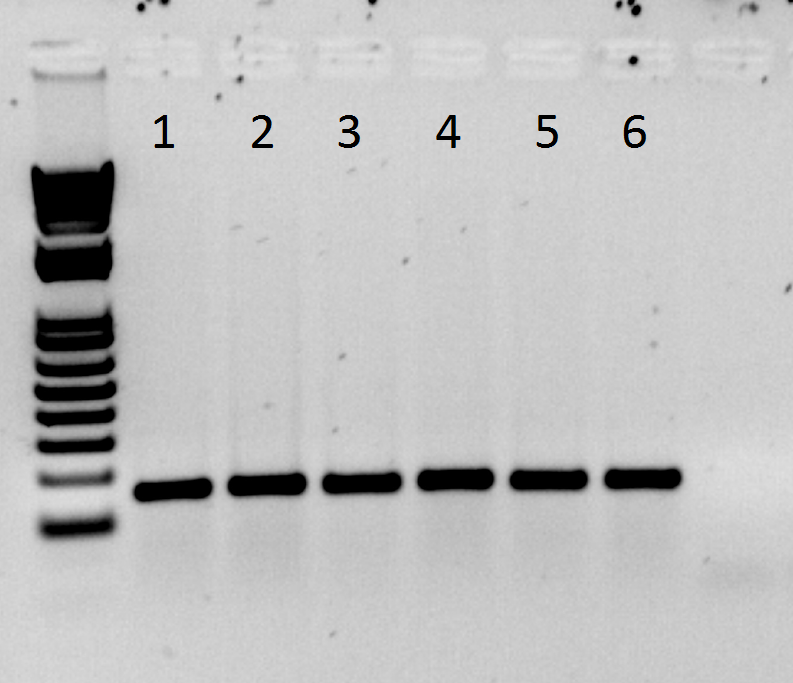


**Figure S12.** cDNA validation excludes cDNA synthesis problems. Donor numbers as in Table S11, showing amplification in all six samples with a set of exon spanning primers C9orf_exon_F and C9orf_exon_R from Table S12.

**A**


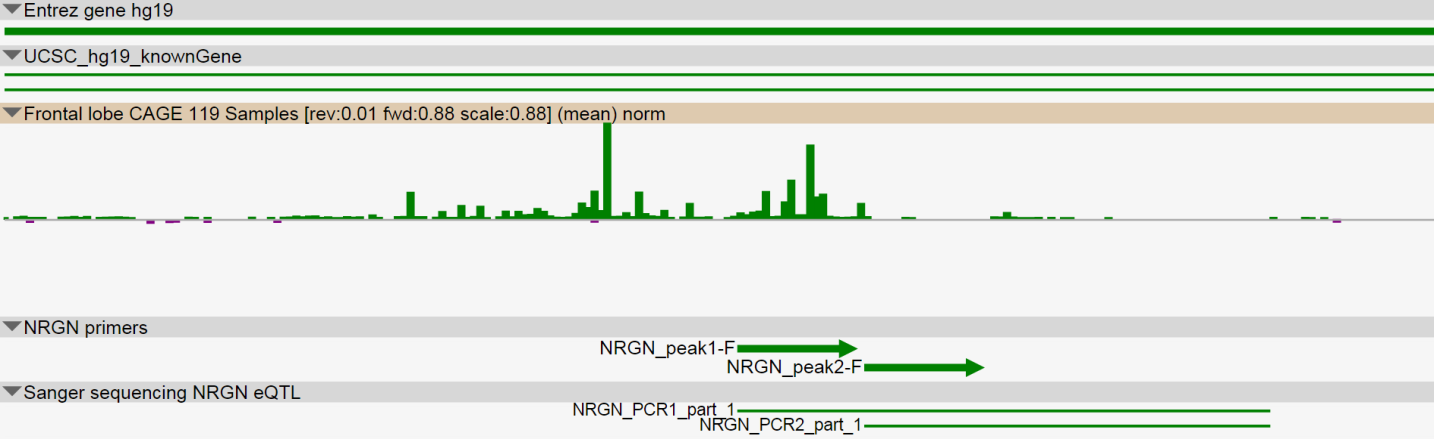


**B**


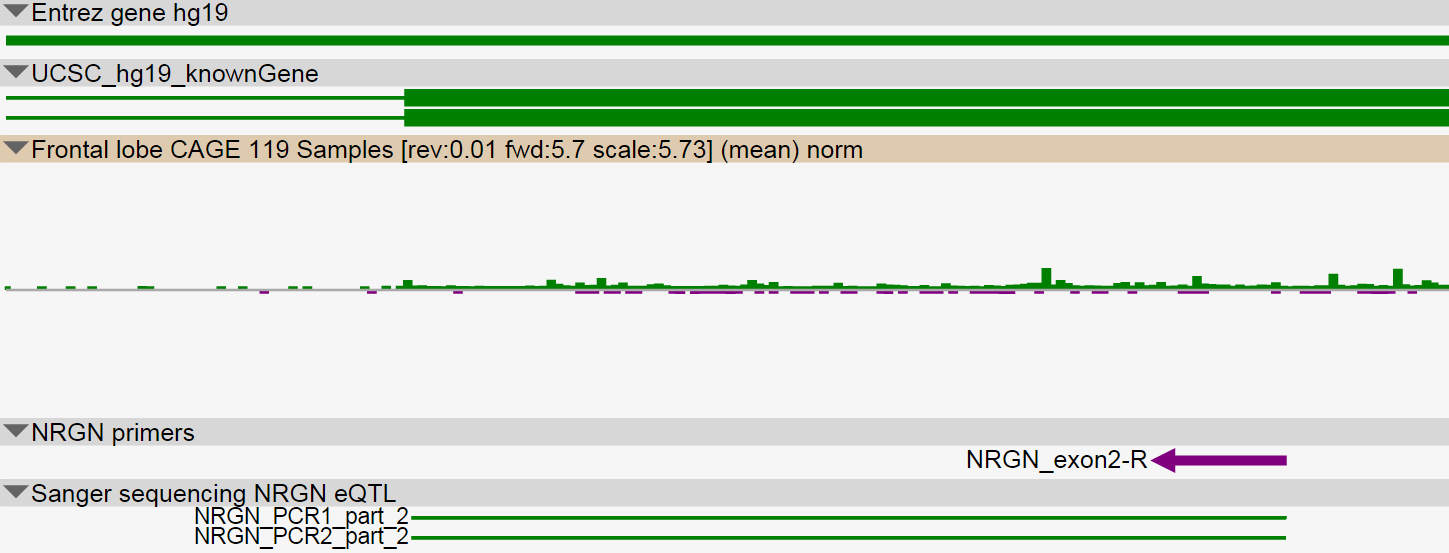


**Figure S13.** Sanger sequencing confirms presence of additional intragenic *NRGN* transcript. Sanger sequencing was performed of two PCR products with primer pairs: NRGN_peak1-F + NRGN_exon2-R and NRGN_peak2-F + NRGN_exon2-R from Table S12 and DNA band are shown in Figure S8. **A** Primers designed directly after the CAGE-cluster NRGN_tss4 show the first part of the transcript. **B** Here the second part of the PCR product is visible and it shows that this transcript uses the same exon 2 boundary as the annotated *NRGN* transcript.


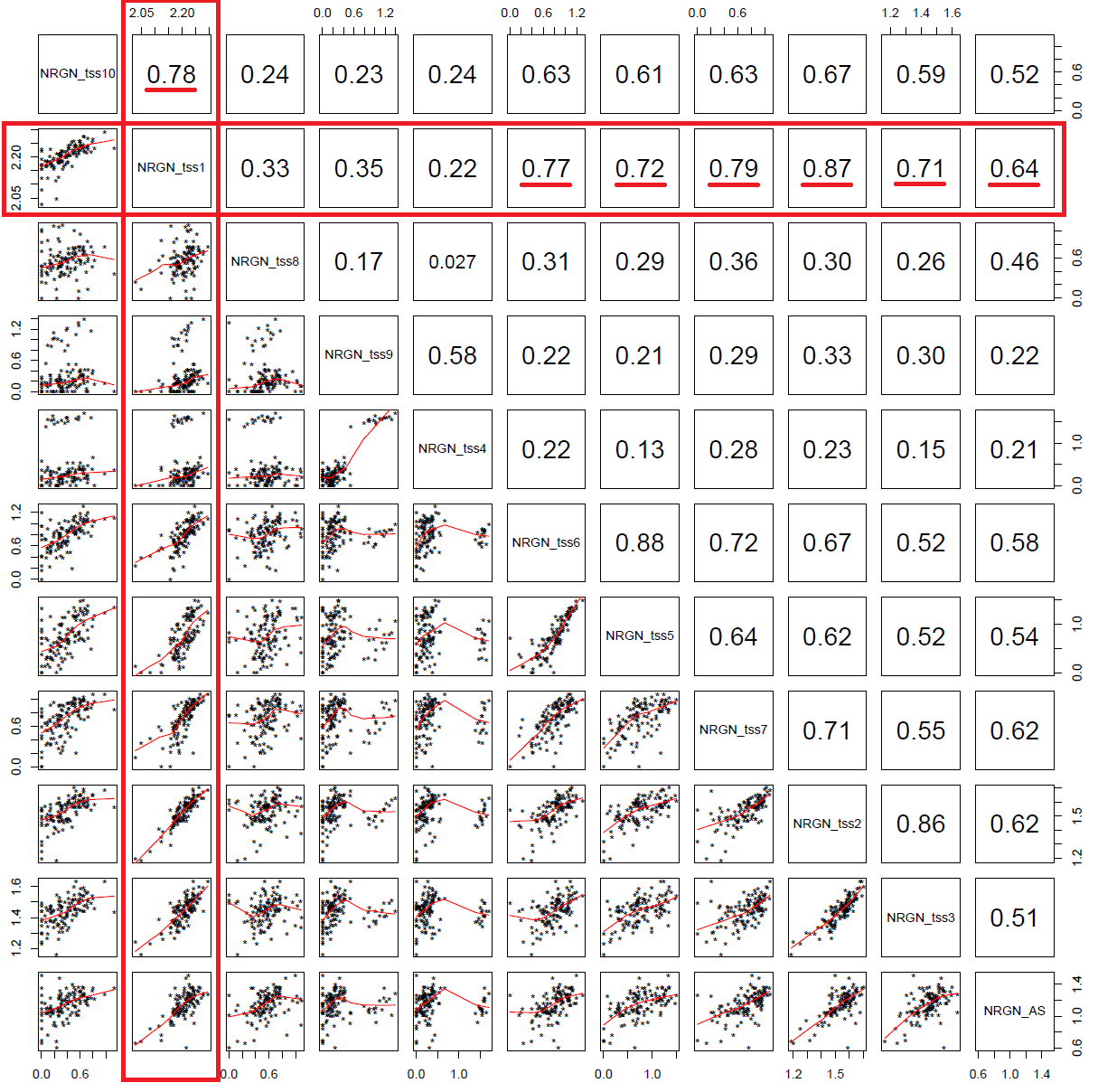


**Figure S14.** Correlations of the intragenic NRGN CAGE-clusters. Spearman correlations between the main TSS of *NRGN* (NRGN_tss1) and other TSS in *NRGN* locus are *highlighted* inside the *red lines*. All show high correlation (>0.64), except three CAGE-clusters: NRGN_tss8 (antisense, low expressed), NRGN_tss9, and NRGN_tss4 (due to eQTL effect).

**Table S2.** Concordance rates between genotyping platforms. High concordance rates were observed between all platforms. na = not applicable

|  | **BeadChip data** | **Imputed** | **Exome sequencing** | **CAGEseq** |
| --- | --- | --- | --- | --- |
| BeadChip data | na | na | >97.5 % | >94.5 % |
| Imputed | na | na | >95.7 % | >91.2 % |
| Exome | >97.5 % | >95.7 % | na | >90.4 % |
| CAGEseq | >94.5 % | >91.2 % | >90.4 % | na |

**Table S3.** External eQTL datasets used for validation.

| **First author** | **Tissue/cell type** | **Technique** | **Included individuals** |
| --- | --- | --- | --- |
| Ramasmamy et al. | Brain | Microarray | 134 |
| Gibbs et al. | Brain | Microarray | 150 |
| Heizen et al. | Brain | Microarray | 93 |
| Myers et al. | Brain | Microarray | 193 |
| Bryois et al. | Lymphoblastoid cell lines | Microarray | 869 |
| Zeller et al. | Monocytes | Microarray | 1490 |
| GTEx brain | Brain frontal cortex | RNAseq | 92 |
| GTEx brain | Brain cortex | RNAseq | 96 |
| GTEx brain | Brain anterior cingulate cortex | RNAseq | 72 |

**Table S4.** Additional included samples for experimental eQTL validation. The brain tissues were obtained from the Dutch Brain Bank. Four individuals were diagnosed with frontotemporal dementia (FTD) and two had no neurological symptoms. CAGEseq expression values were obtained from an internal CAGEseq expression database that includes the samples used for eQTL validation. FTD = frontotemporal dementia; tpm = tags per million.

| **ID (internal)** | **Donor** | **Brain region** | **Note** | **Expression level (tpm)** |
| --- | --- | --- | --- | --- |
| 11040 | 1 | Frontal cortex | control | 47 |
| 92017 | 2 | Frontal cortex | FTD | 0.45 |
| 07106 | 3 | Frontal cortex | FTD | 18 |
| 10166 | 4 | Frontal cortex | FTD | 3.4 |
| 12005 | 5 | Frontal cortex | Control | 0.23 |
| 00136 | 6 | Frontal cortex | FTD | 0.17 |

**Table S5.** Primer sequences used for experimental eQTL validation. C9orf_exon primers were used as cDNA quality control.

| **Name** | **Sequence ‘5 – ‘3** | **Annealing temperature** |
| --- | --- | --- |
| NRGN_peak1-F | CGAGGGGTTTGCTGTAAAGG | 58 °C |
| NRGN_peak2-F | TGACTCGGTATGAAAGCGCC | 58 °C |
| 3RACE_NRGN1-F | AGGTGACTCGGTATGAAAGCG | 58 °C |
| 3RACE_NRGN2-F | TCCTGTAAAGGTGACTCGGT | 58 °C |
| 3RACE_NRGN3-F | GAAACCTGGGTACTGCAAGTG | 58 °C |
| 3RACE_NRGN4-F | TACTGCAAGTGCCCAGCGCC | 58 °C |
| NRGN_exon2-R | TATCTTCTTCCGCGCCATGT | 58 °C |
| C9orf_exon_F | GCCTTTTTCTGACTCCAGCA | 58 °C |
| C9orf_exon_R | TGTGTGTGGTGGGATATGGA | 58 °C |
| PARK16_F1 | AGTAGGAGGAGTGACGGGAACC | 58 °C |
| PARK16_R2 | CGAACTCAGCCAGGGCTGTAGTAT | 58 °C |

**Table S6.** Primer sequences used for genotyping of additional samples.

| **Name** | **Sequence ‘5 – ‘3** | **Annealing temperature** |
| --- | --- | --- |
| rs320881_F | CGGTTACCAATCTGAAACCCTG | 58 °C |
| rs320881_R | ACTACCTCCTTCCTCCCACA | 58 °C |
| rs35306015_F | TGGCGAGGAAAGGAAGTGTC | 58 °C |
| rs35306015_R | CTACAGGGCAGGCATTCACA | 58 °C |

**Table S10.** Genetic location eQTL variant enrichments. Variants located in “gene-associated” portions of the genome (e.g. 5' UTRs, exons, and regions upstream to annotated genes) are more likely to be an eQTL compared to the genome average (represented by “All variants”). The opposite trend is observed by variants located in intergenic regions.

|  | **All variants (%)** | **All eQTL (%)** | **All sentinel eQTLs (%)** |
| --- | --- | --- | --- |
| Exonic | 0.6 | 1.7 | 5.4 |
| Intergenic | 58.5 | 38.6 | 34.0 |
| Intronic | 35.5 | 46.9 | 39.9 |
| ncRNA_exonic | 0.2 | 1.0 | 2.0 |
| ncRNA_intronic | 3.0 | 6.3 | 4.2 |
| Downstream | 0.6 | 1.5 | 1.1 |
| Upstream | 0.6 | 1.6 | 5.4 |
| UTR3 | 0.8 | 1.7 | 3.1 |
| UTR5 | 0.2 | 0.6 | 4.6 |
| Other | 0.0 | 0.1 | 0.3 |
|  | 100 | 100 | 100 |

**Table S11.** Functional element eQTL variant enrichments. Sentinel eQTL variants are more likely to be located in functional elements compared to the genome average (represented by “All variants”).

| **Feature** | **All variants (%)** | **eQTL variants (%)** | **Sentinel variants (%)** |
| --- | --- | --- | --- |
| H3K27ac | 5.1 | 11.4 | 30.2 |
| DHS-brain | 8.1 | 15.6 | 36.1 |
| DHS-general | 39.9 | 47.3 | 64.6 |
| Any | 40.5 | 49.4 | 67.3 |
